# Supplementary material for: Continuous map of early hematopoietic stem cell differentiation across human lifetime
Source: Nat Commun. 2025 Mar 7;16:2287. doi: 10.1038/s41467-025-57096-y (PMC11889232; doi:10.1038/s41467-025-57096-y)
Supplement: Supplementary file 1 — Supplementary Information [file 41467_2025_57096_MOESM1_ESM.pdf]

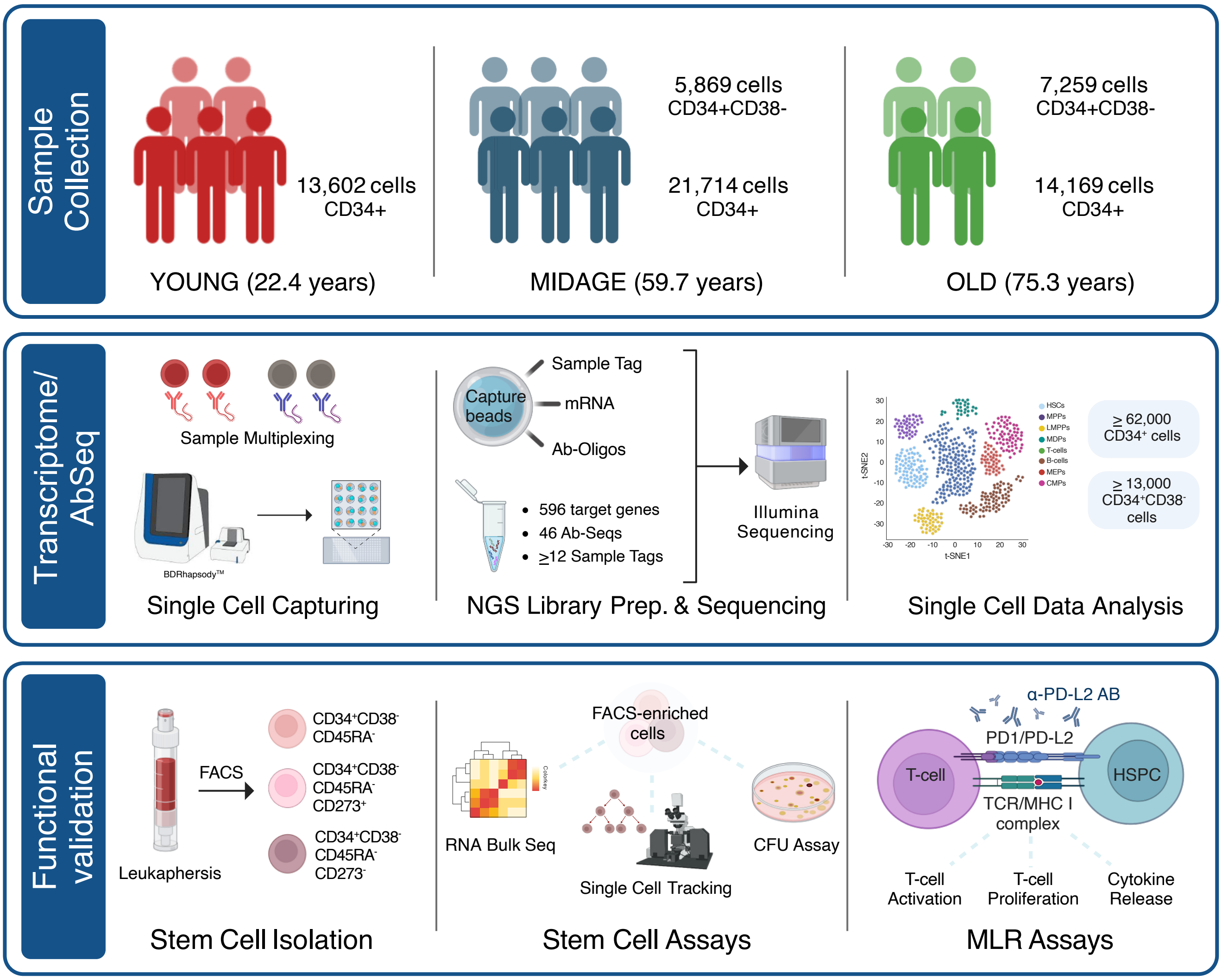

Supplementary Figure S1. Graphical abstract of the workflow.
HSPCs were isolated from 15 donors of three different age groups (Sample collection). After sample multiplexing and antibody-based sequencing (AbSeq) staining for surface proteins, transcriptome analysis was performed using the BD Rhapsody platform. Transcriptome/AbSeq data analysis was performed on 62,277 cells passing quality control. CD273/PD-L2 was identified to be highly expressed on immature stem cells, and functional validation including the interplay with allogeneic T-cells was performed (Functional validation).Created in BioRender. Rieger, A. (2025) https://BioRender.com/n82a359.

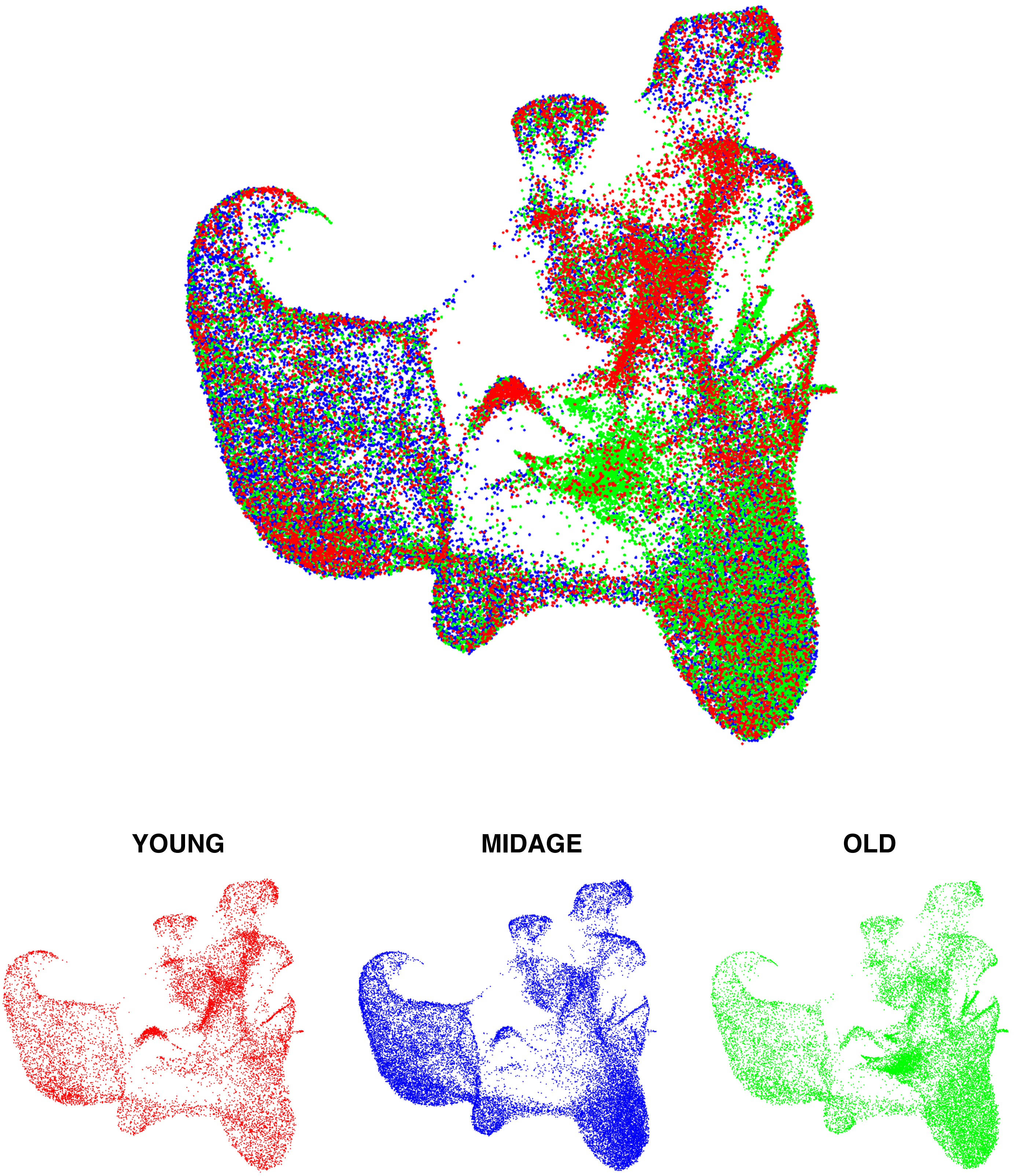

Supplementary Figure S2. Distribution of CD34+ HSPCs from three age groups after batch effect correction.
62,277 single cells from 15 donors of three age groups projected on UMAP after CCA batch effect correction. Superimposed and isolated UMAP plots are shown.

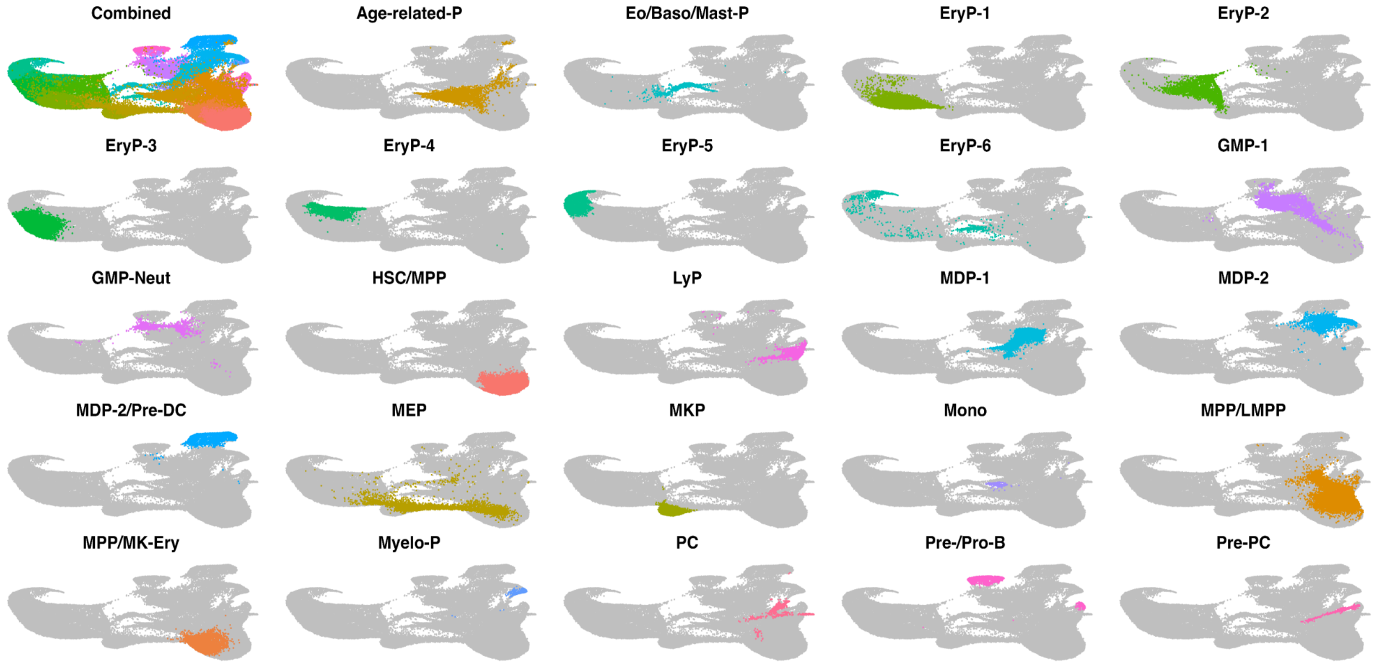

Supplementary Figure S3. Visualization of individual clusters.
UMAP plots display separated clusters of CD34^{+} HSPCs from Figure 1a.

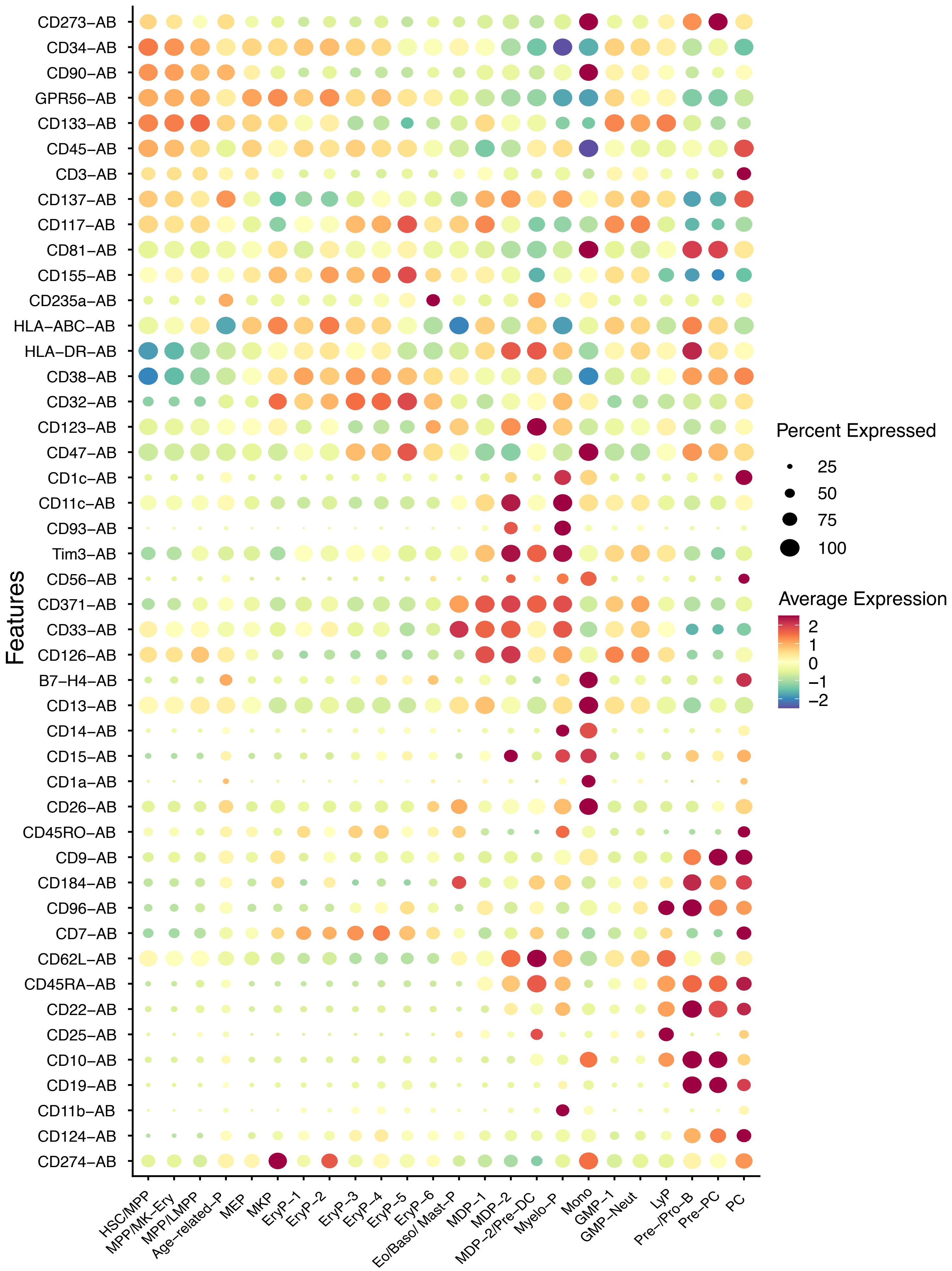

Supplementary Figure S4. Surface protein expression of CD34⁺ HSPC clusters.
Dot-plot visualization of the surface protein expression of 46 AbSeq markers in UMAP clusters from CD34⁺ HSPCs shown in Figure 1a.

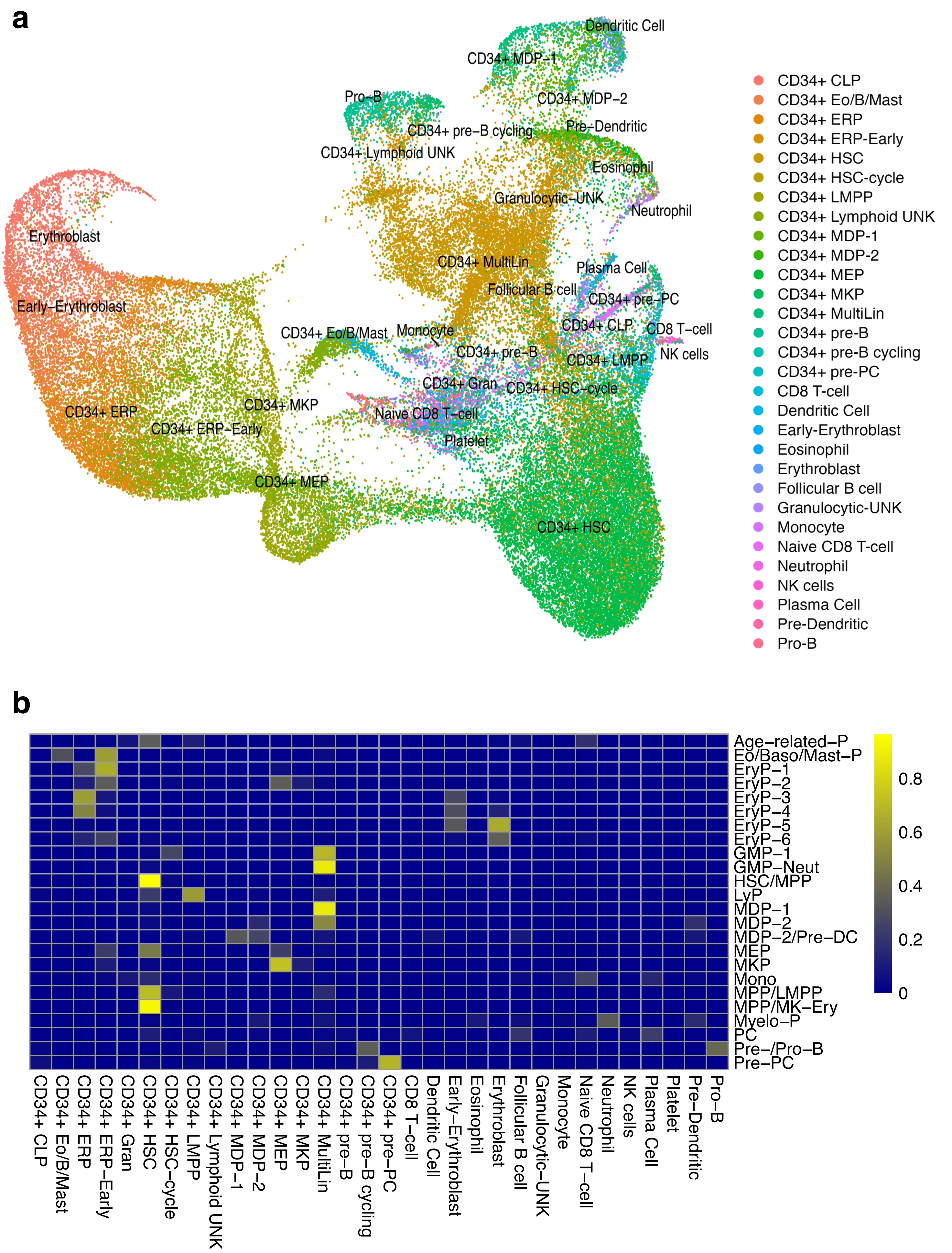

Supplementary Figure S5. Unsupervised cell type annotation by cell label transfer. a) Cell label transfer was performed using the published dataset from Triana *et al.* Nat Immunol 2021. b) Correlation matrix: X axis = predicted labels from cell label transfer, Y-axis = our manual annotation

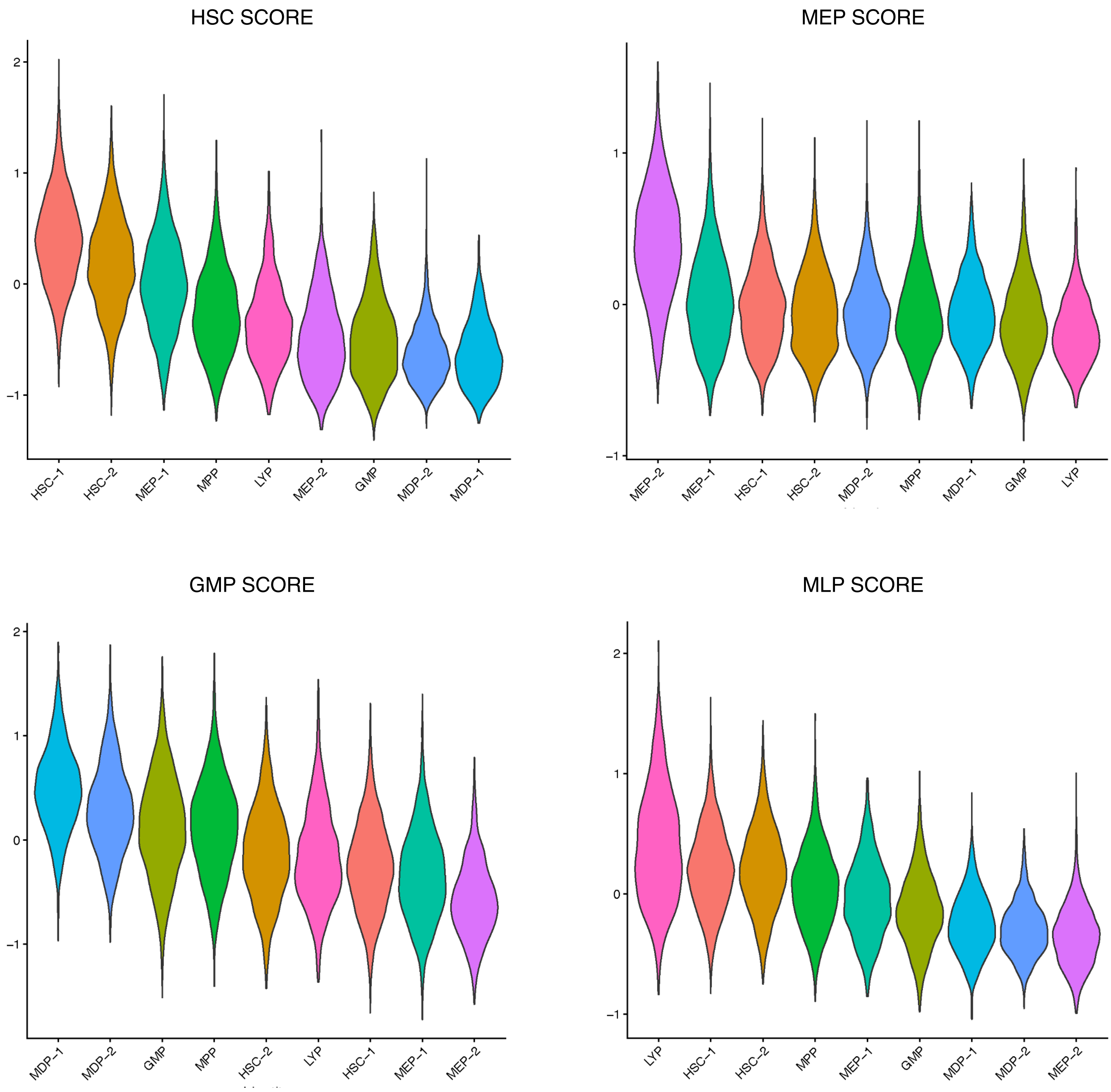

Supplementary Figure S6. Cell population scores of clusters from immature HSPCs.
Violin plots showing HSC and progenitor cell scores calculated for each cell analyzed in the re-clustered immature population (n = 21,395 cells) shown in Figure 2a using the published gene sets by Mende *et al.* Blood 2022.

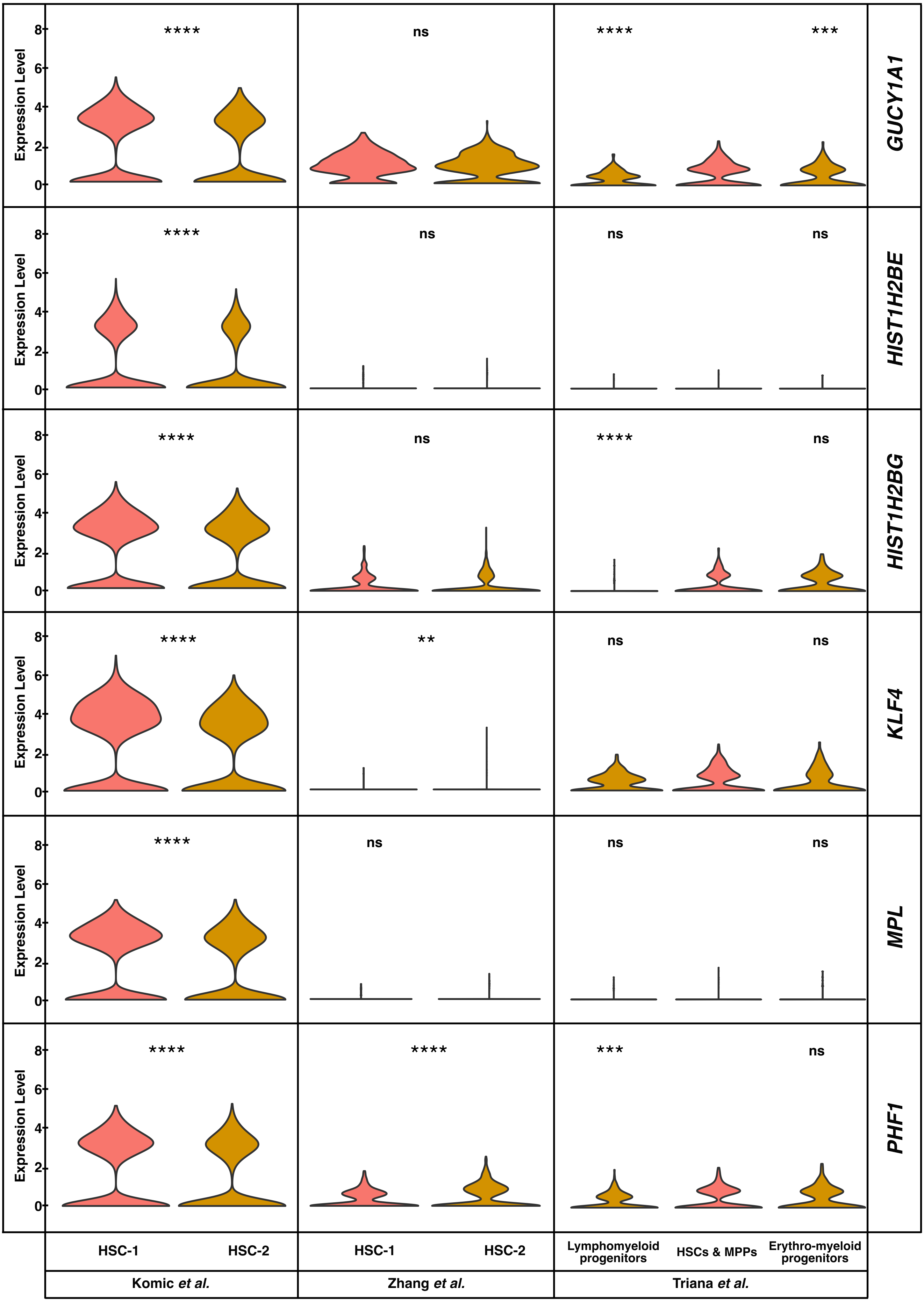

Supplementary Figure S7. Gene expression of HSPC genes identified in our study (Komic *et al.*) and confirmed/compared to the studies of Zhang *et al.* and Triana *et al.* Statistics was calculated by Wilcoxon Rank Sum test; **, $p<0.01$; ***, $p<0.001$, ****, $p<0.0001$; ns, not significant.

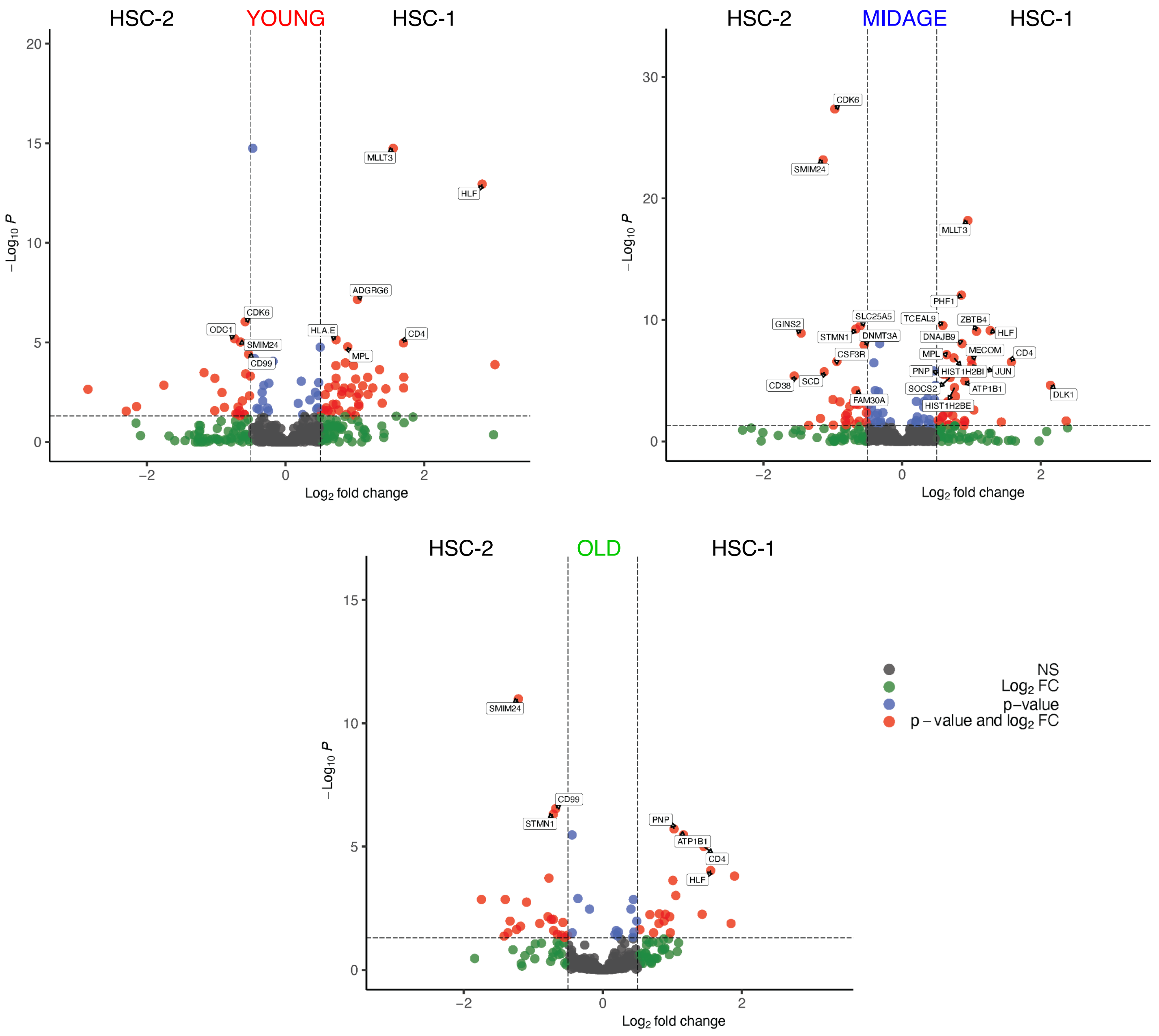

Supplementary Figure S8. Differential gene expression of HSC-1 and HSC-2 cells according to age.

Volcano plots depict the results of DESeq2 analysis on pseudobulk data, comparing HSC-1 and HSC-2 cluster cells, within each age group. A detailed gene list is shown in Supplementary Tables 9-11.

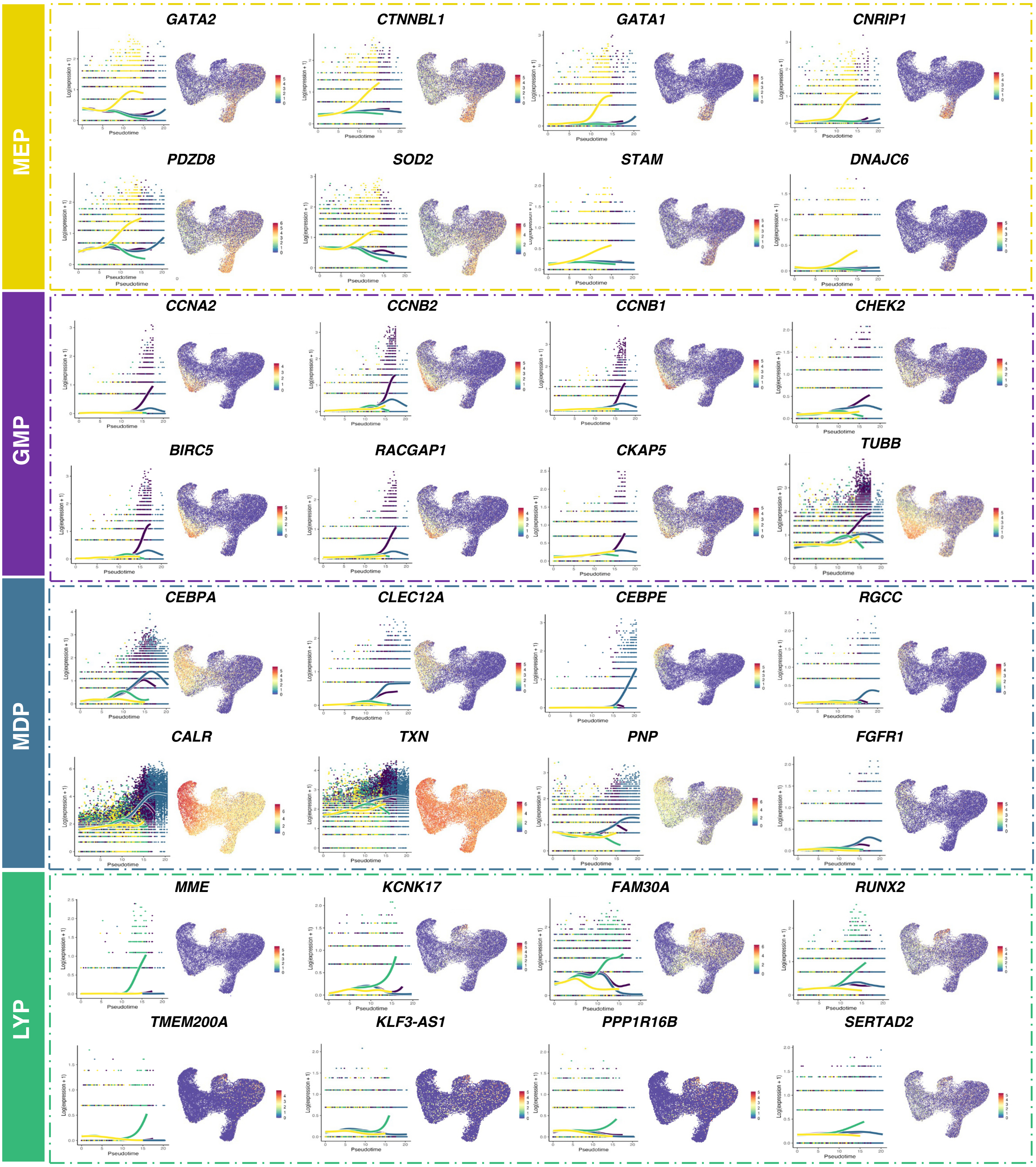

Supplementary Figure S9. Continuous representation of gene expression by tradeSeq regression model based on lineage trajectories.
Showcases genes that are specifically expressed in one trajectory: MKP/ERP (yellow), GMP (violet), MDP (blue) and LYP (green). An extended list of genes is provided in the Supplementary Material.

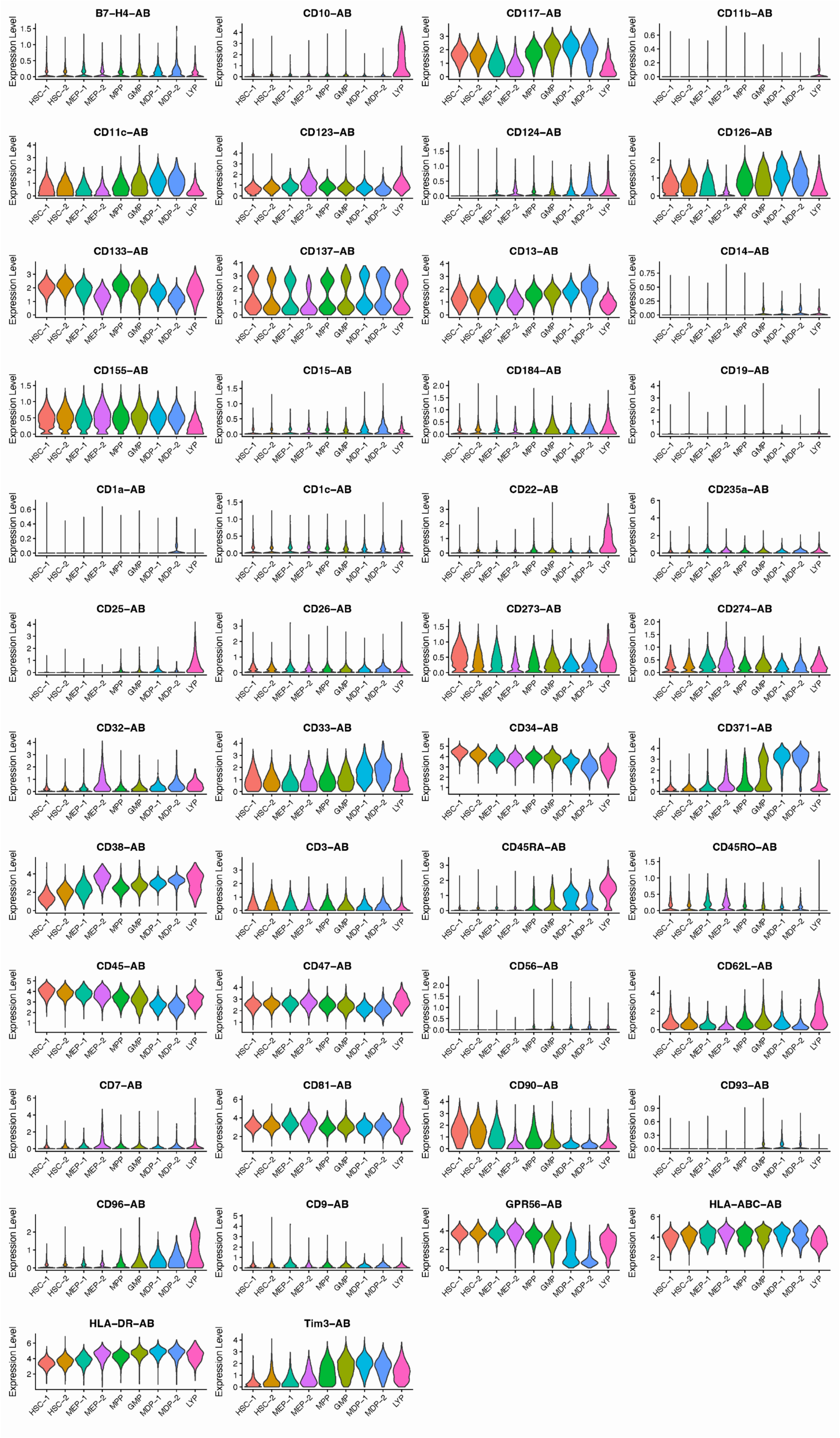
B7−H4−AB
CD10−AB
CD117−AB
CD11b−AB
CD11c−AB
CD123−AB
CD124−AB
CD126−AB
CD133−AB
CD137−AB
CD13−AB
CD14−AB
CD155−AB
CD15−AB
CD184−AB
CD19−AB
CD1a−AB
CD1c−AB
CD22−AB
CD235a−AB
CD25−AB
CD26−AB
CD273−AB
CD274−AB
CD32−AB
CD33−AB
CD34−AB
CD371−AB
CD38−AB
CD3−AB
CD45RA−AB
CD45RO−AB
CD45−AB
CD47−AB
CD56−AB
CD62L−AB
CD7−AB
CD81−AB
CD90−AB
CD93−AB
CD96−AB
CD9−AB
GPR56−AB
HLA−ABC−AB
HLA−DR−AB
Tim3−AB
Expression Level
HSC−1
HSC−2
MEP−1
MEP−2
MPP
GMP
MDP−1
MDP−2
LYP

Supplementary Figure S10. Surface protein expression of immature HSPC clusters. Violin-plot visualization of the surface protein expression of 46 AbSeq markers in UMAP clusters from re-clustered immature HSPCs shown in Figure 2a.

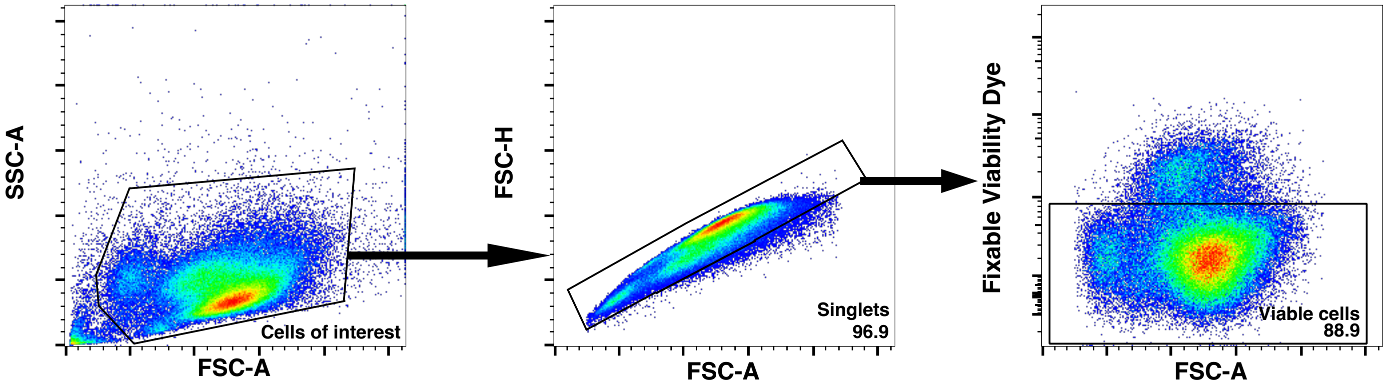

Supplementary Figure S11. Gating strategy to enrich for viable singlet cells in flow cytometry analysis.

All samples were first gated using FSC-A/SSC-A to exclude debris, followed by FSC-H/FSC-A to identify singlet cells and by using a fixable viability dye to exclude dead cells. The displayed example represents CD34 MACS-enriched mPB. All relevant subsequent gating strategies are shown in the main or supplementary figures.

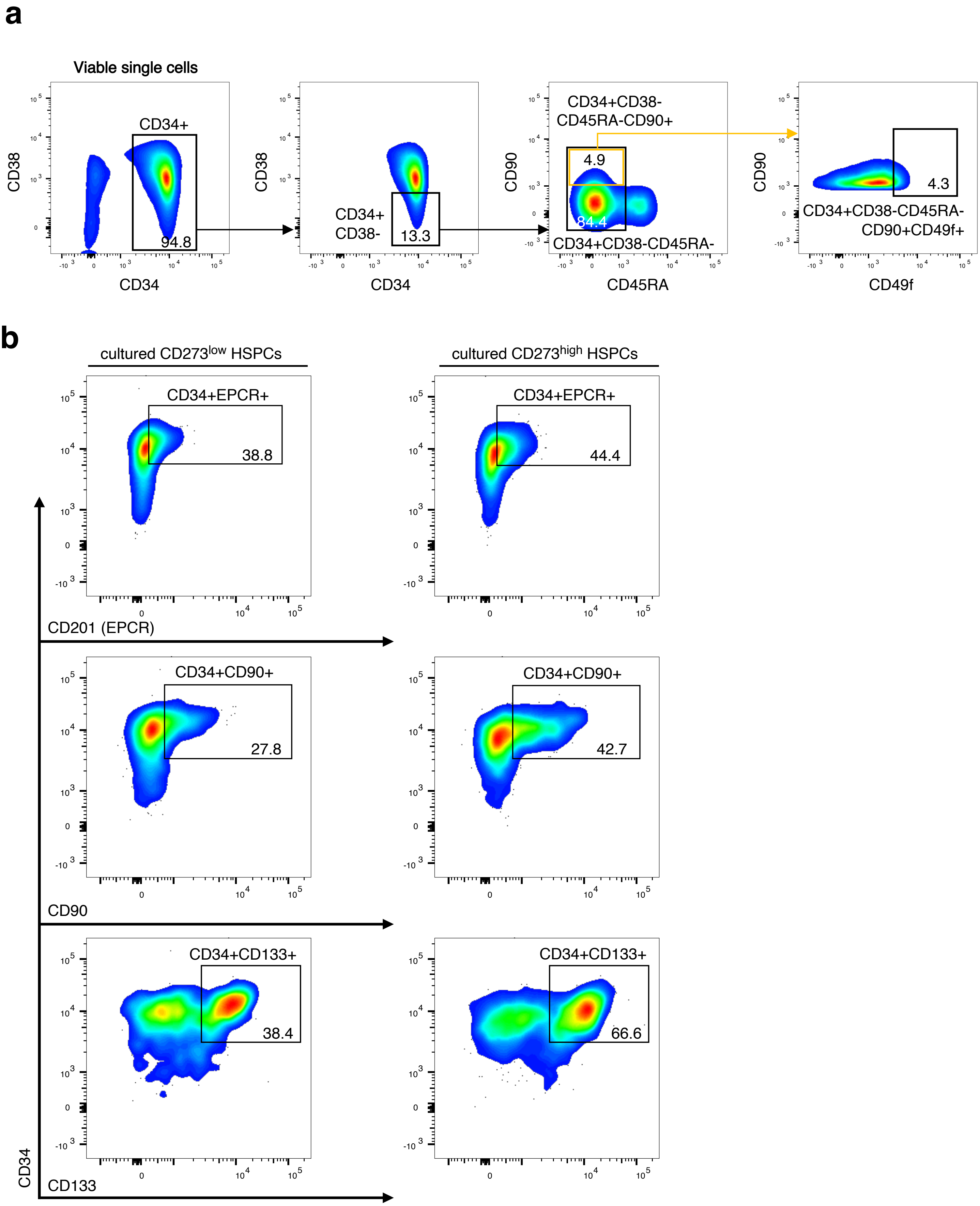

Supplementary Figure S12. FACS gating of HSPC populations in functional analyses.
a) Representative flow cytometry plots for analyzing CD273 expression levels on defined HSPC subpopulations with their percentages. b) Representative plots of *in vitro*-cultured CD273^{low} and CD273^{high} sorted HSPCs. Gates show CD34+ cells with expression of CD201(EPCR), CD90 and CD133 and their percentages.

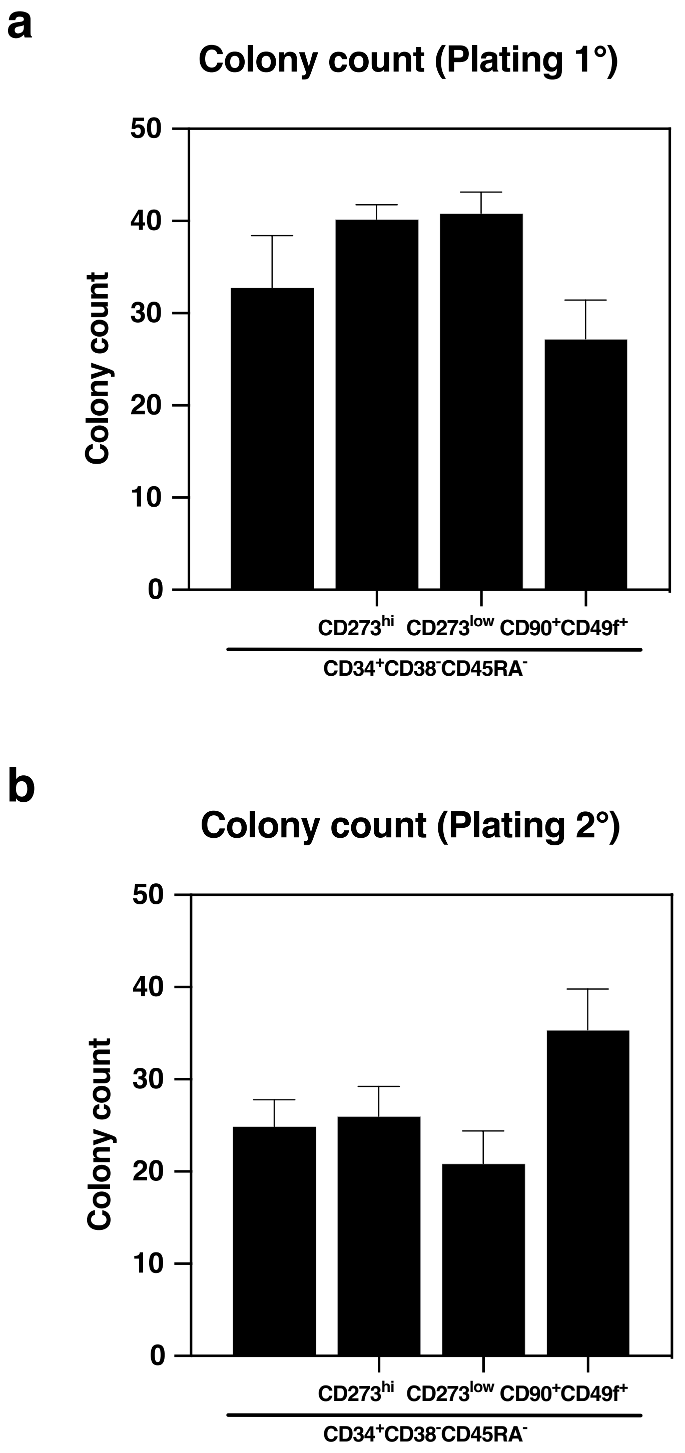

Supplementary Figure S13. Number of colonies determined by colony-forming unit assay (CFU).
a) FACS-sorted cells of indicated subpopulations after 14 days of culture. b) Replating of primary CFU and colony scoring after 14 days.

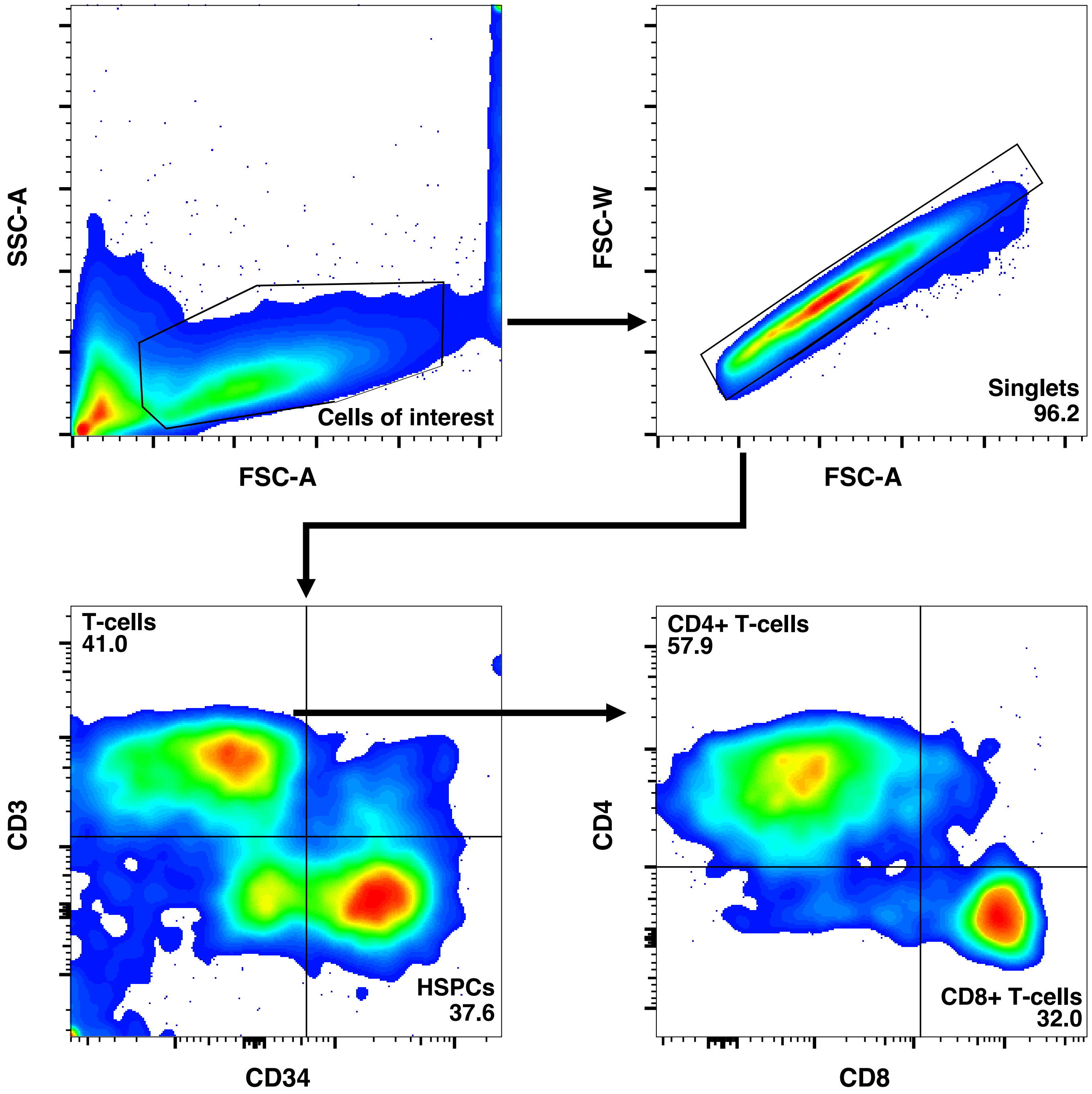

Supplementary Figure S14. Gating strategy for T-cells in co-culture experiments.
All samples were FSC-A and SSC-A gated for debris exclusion, followed by FSC-W/FSC-A gating to select singlet cells. T-cells were distinguished from HSPCs by CD3 expression. Subsets of T-cells were gated based on CD4 or CD8 expression.

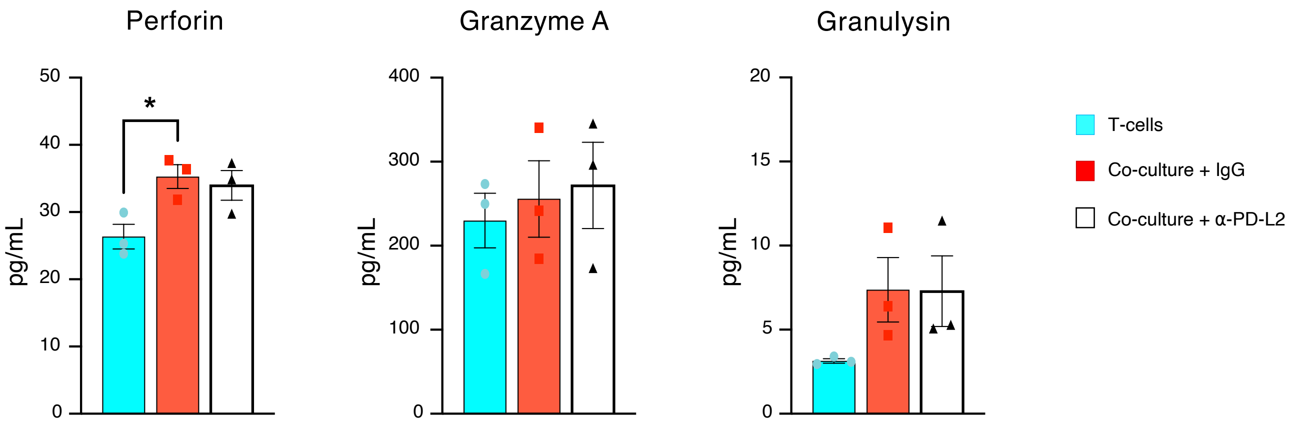

Supplementary Figure S15. Cytokine release of co-culture of allogeneic HSPCs and T-cells. T-cells were co-cultured with allogeneic HSPCs in the presence of either PD-L2 blocking antibody (black) or an isotype control antibody (red) for 72 h, and compared to a T-cell mono-culture (blue). Cytokine-bead array assay of co-culture and T-cell mono-culture supernatants (n=3). ANOVA with Tukey's multiple comparisons test. Bars represent the mean with SEM. *, $p<0.05$.

References

Mende, N. et al. Unique molecular and functional features of extramedullary hematopoietic stem and progenitor cell reservoirs in humans. Blood 139, 3387–3401; 10.1182/blood.2021013450 (2022).

Triana, S. et al. Single-cell proteo-genomic reference maps of the hematopoietic system enable the purification and massive profiling of precisely defined cell states. Nature immunology 22, 1577–1589; 10.1038/s41590-021-01059-0 (2021).

Zhang, X. et al. An immunophenotype-coupled transcriptomic atlas of human hematopoietic progenitors. Nature Immunology 25, 703–715; 10.1038/s41590-024-01782-4 (2024).
