# Supplementary material for: Continuous map of early hematopoietic stem cell differentiation across human lifetime
Source: Nat Commun. 2025 Mar 7;16:2287. doi: 10.1038/s41467-025-57096-y (PMC11889232; doi:10.1038/s41467-025-57096-y)
Supplement: Supplementary file 2 — Description of Additional Supplementary Files [file 41467_2025_57096_MOESM2_ESM.pdf]

Description of Additional Supplementary Files

Supplementary Data 1. BD Rhapsody gene panel and primer sequences for PCR1 and PCR2

Supplementary Data 2. Oligonucleotide-conjugated antibodies (AbSeq) used in the proteo-transcriptomic single cell sequencing analysis

Supplementary Data 3. Donor information (proteo-transcriptomic sequencing)

Supplementary Data 4. Differential gene expression of CD34+ cells. Significance was calculated by a Wilcoxon Rank Sum test with Bonferroni correction

Supplementary Data 5. Cluster annotation for CD34+ UMAP (manual and cell label transfer from Triana et al.)

Supplementary Data 6. Differential gene expression of immature HSPCs. Significance was calculated by a Wilcoxon Rank Sum test with Bonferroni correction

Supplementary Data 7. DESeq2 pseudobulk results: comparison of HSC-1 (upregulated) vs. HSC-2 (downregulated). Significance was calculated by a two-sided Wald test with Benjamini-Hochberg correction

Supplementary Data 8. Comparison of upregulated marker genes in the most immature cell cluster of three independent datasets (genes identified by FindAllMarkers)

Supplementary Data 9. DESeq2 pseudobulk results: comparison of HSC-1 (upregulated) vs. HSC-2 (downregulated) in YOUNG age group. Significance was calculated by a two-sided Wald test with Benjamini-Hochberg correction

Supplementary Data 10. DESeq2 pseudobulk results: comparison of HSC-1 (upregulated) vs. HSC-2 (downregulated) in MIDAGE group. Significance was calculated by a two-sided Wald test with Benjamini-Hochberg correction

Supplementary Data 11. DESeq2 pseudobulk results: comparison of HSC-1 (upregulated) vs. HSC-2 (downregulated) in OLD age group. Significance was calculated by a two-sided Wald test with Benjamini-Hochberg correction

Supplementary Data 12. Overlapping upregulated genes in HSC-1 vs. HSC-2 in different age groups (see Venn diagram in Figure 2f)

Supplementary Data 13. Continuous representation of gene expression by tradeSeq regression model based on lineage trajectories

Supplementary Data 14. Differential surface protein expression of HSC-1 vs. HSC-2. Significance was calculated by a Wilcoxon Rank Sum test with Bonferroni correction

Supplementary Data 15. Bulk RNA-seq of FACS-sorted HSPCs according to their CD273 expression (CD273high vs CD273low)

Supplementary Data 16. Donor information for functional and molecular validation experiments

Supplementary Data 17. List of antibodies used for FACS sorting and functional validation experiments
