# Supplementary figures and images for: Continuous map of early hematopoietic stem cell differentiation across human lifetime
Source: Nat Commun. 2025 Mar 7;16:2287. doi: 10.1038/s41467-025-57096-y (PMC11889232; doi:10.1038/s41467-025-57096-y)

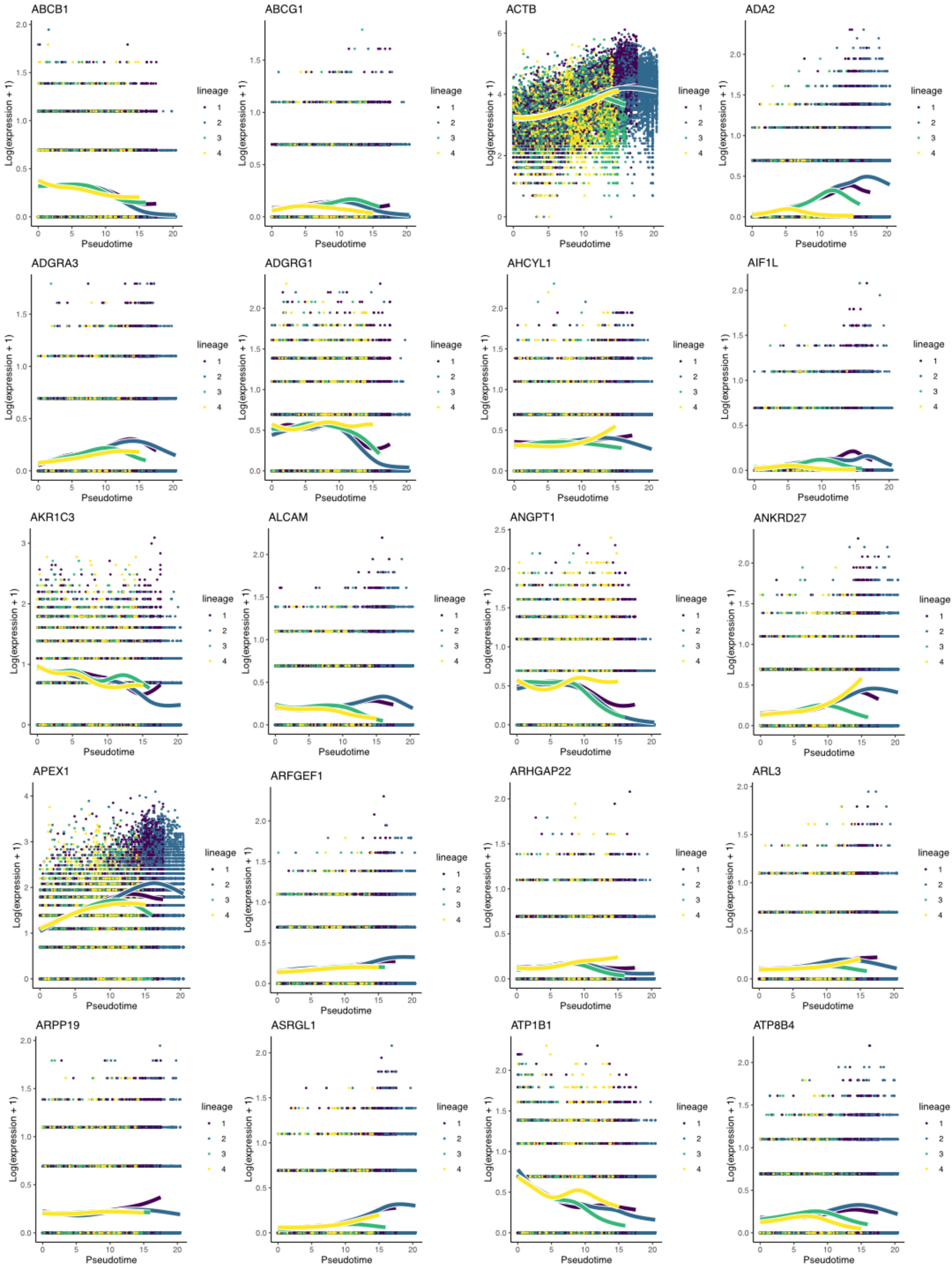

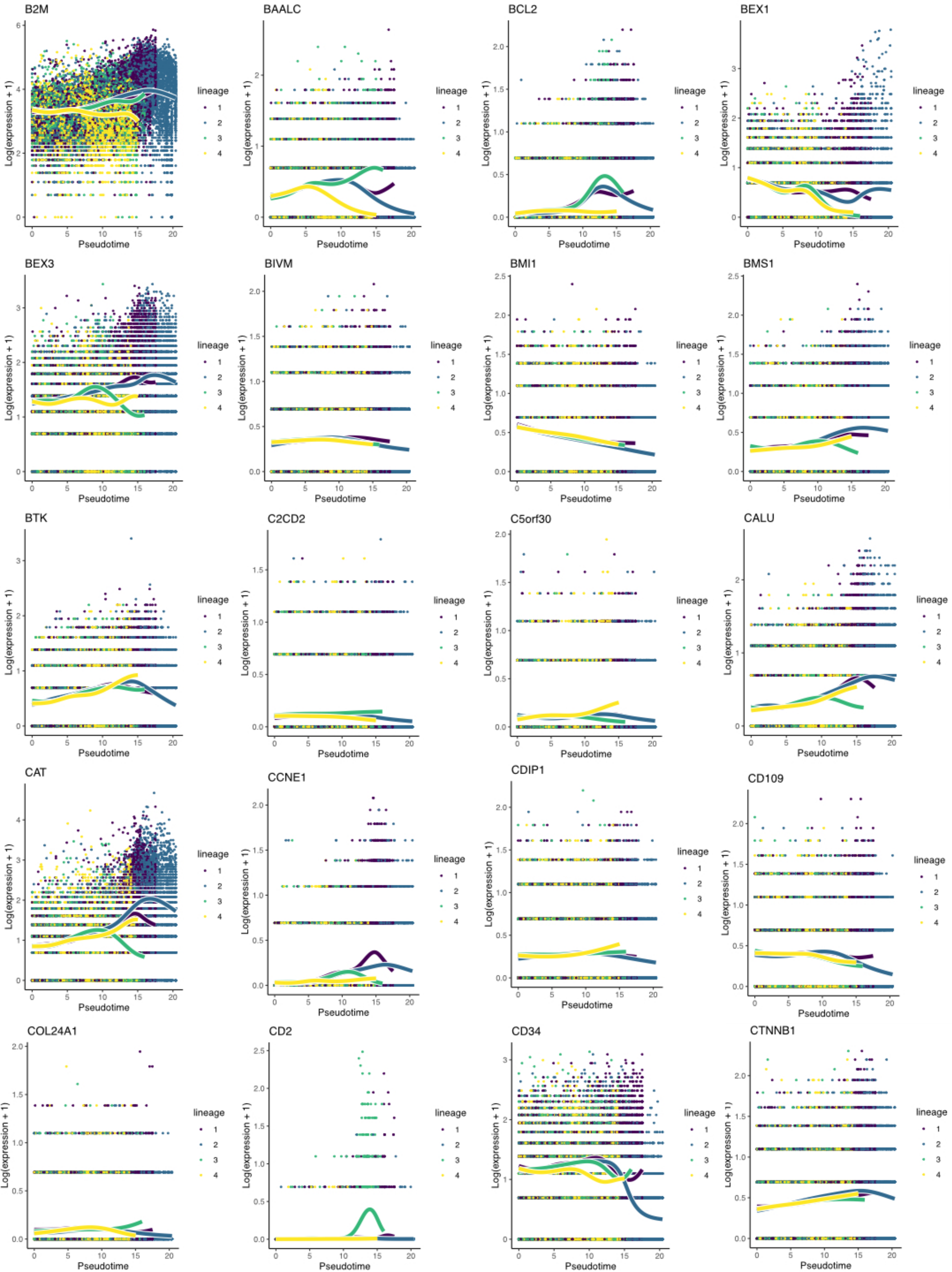

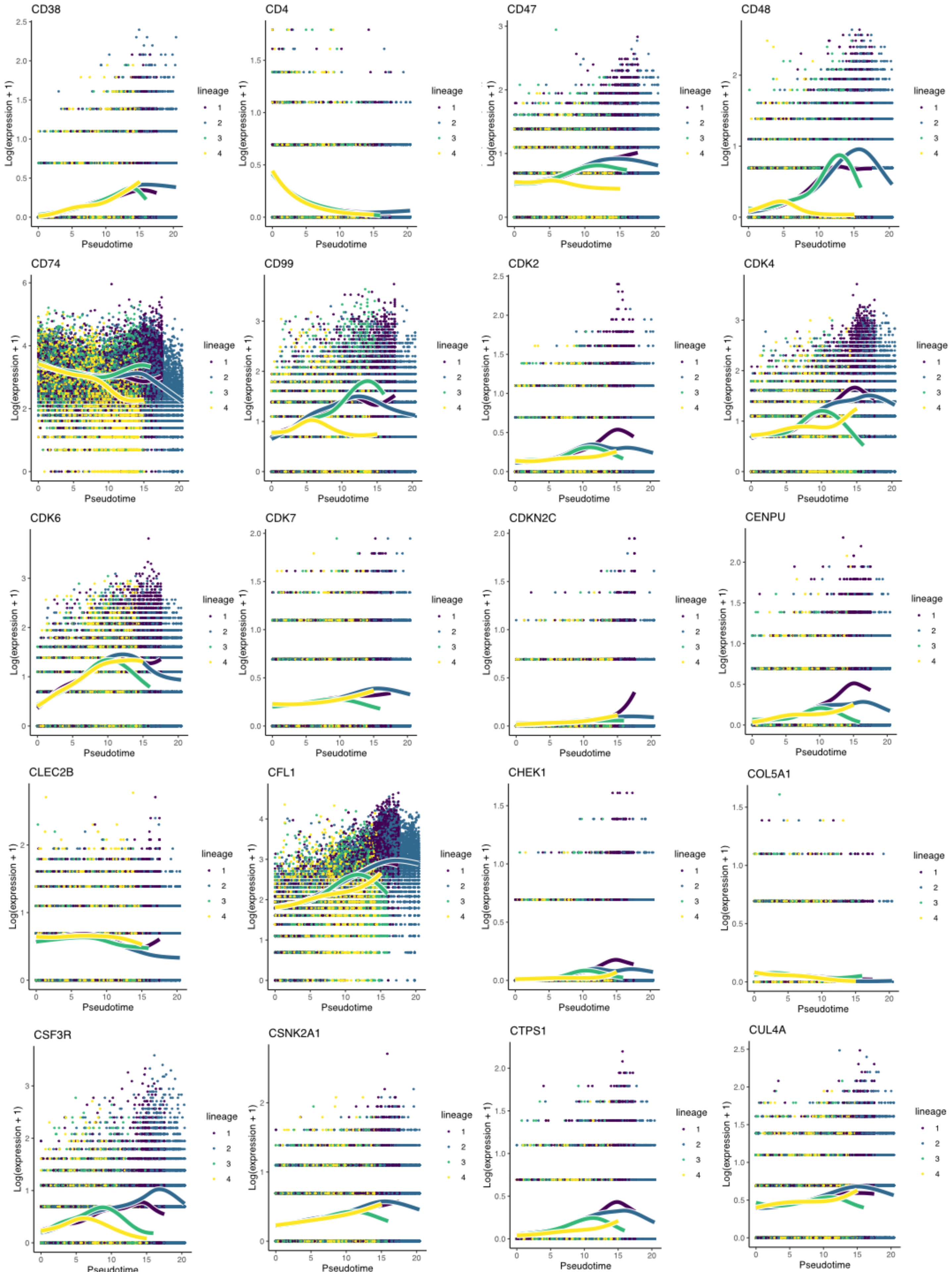

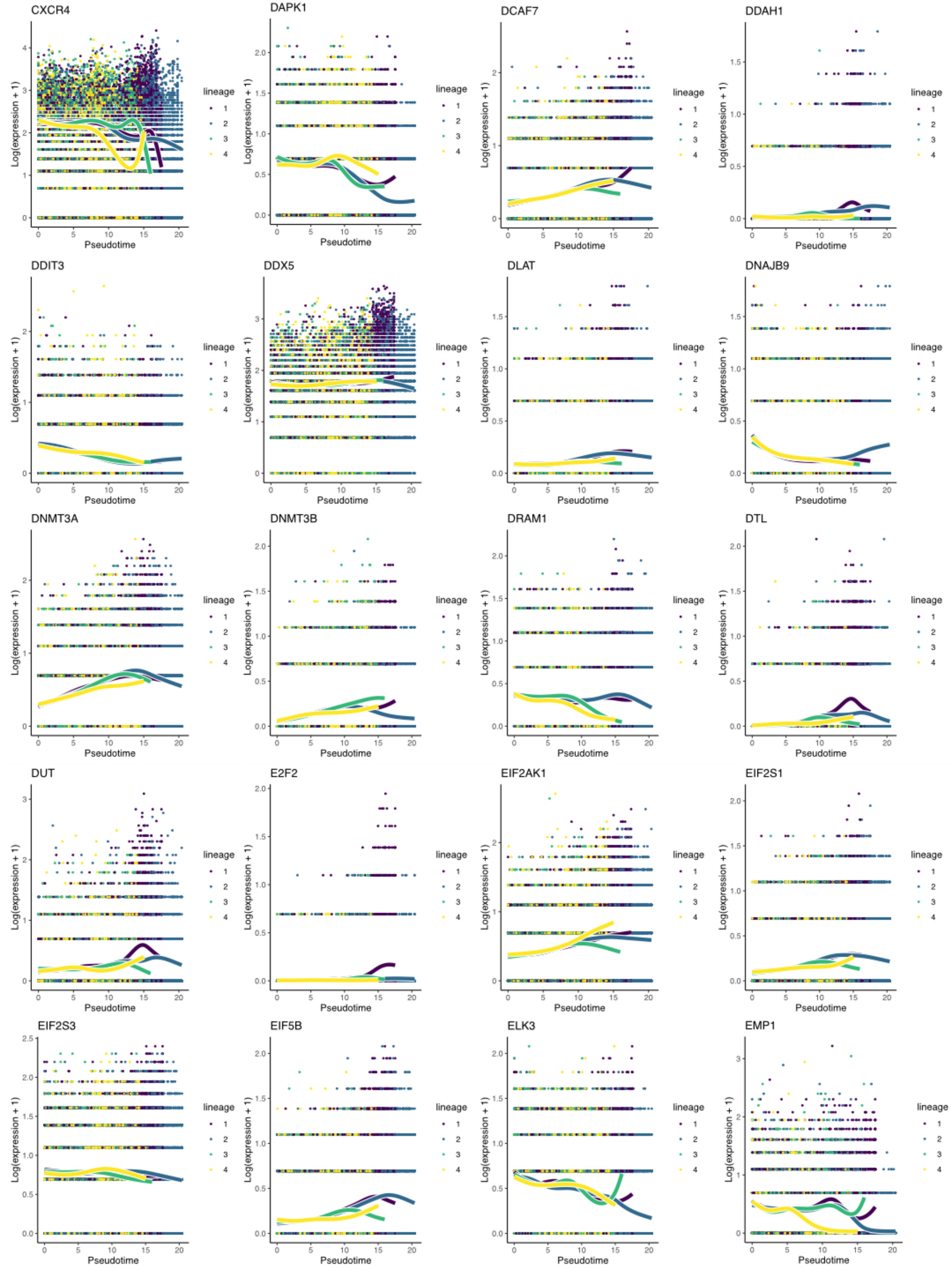

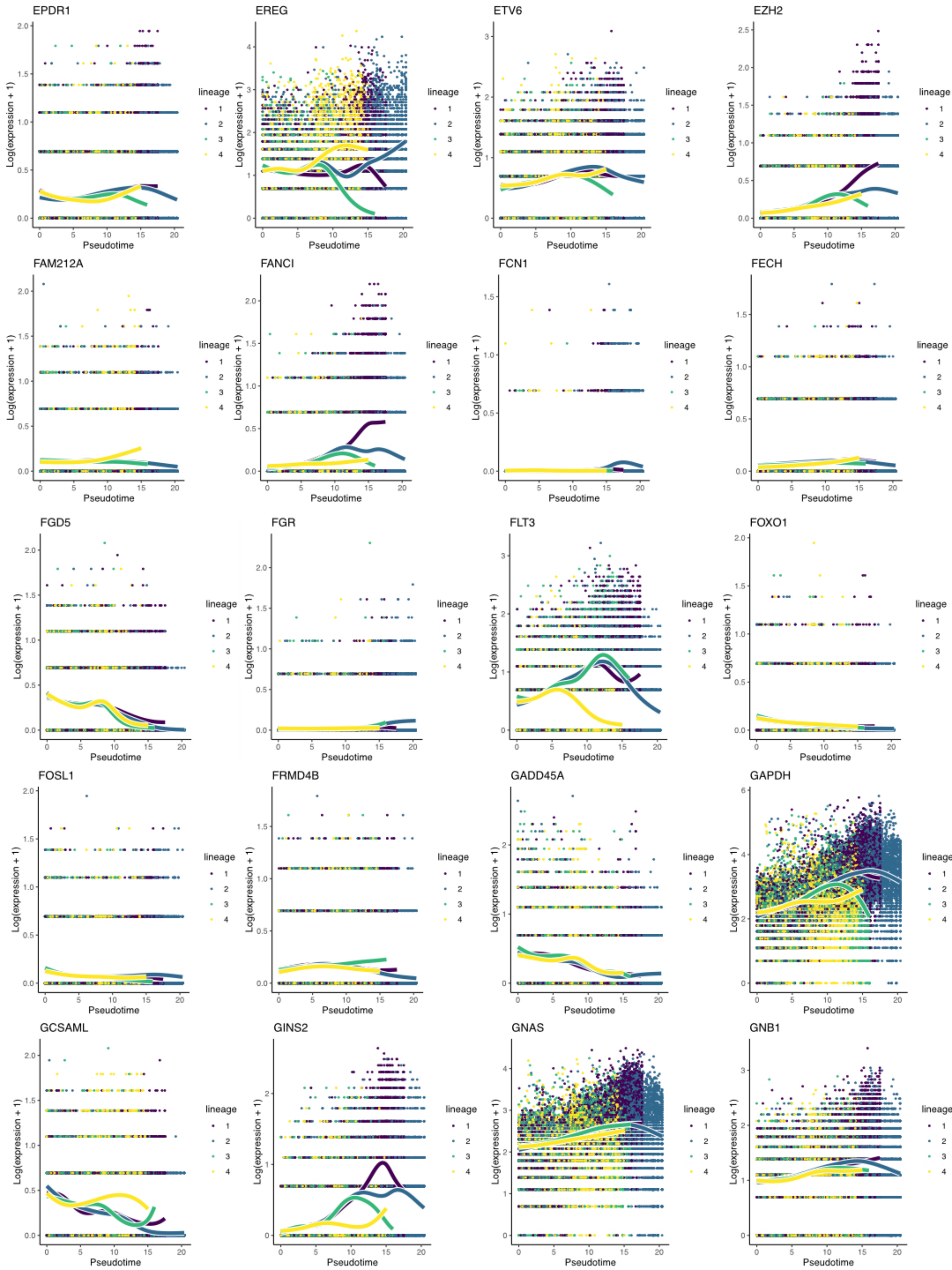

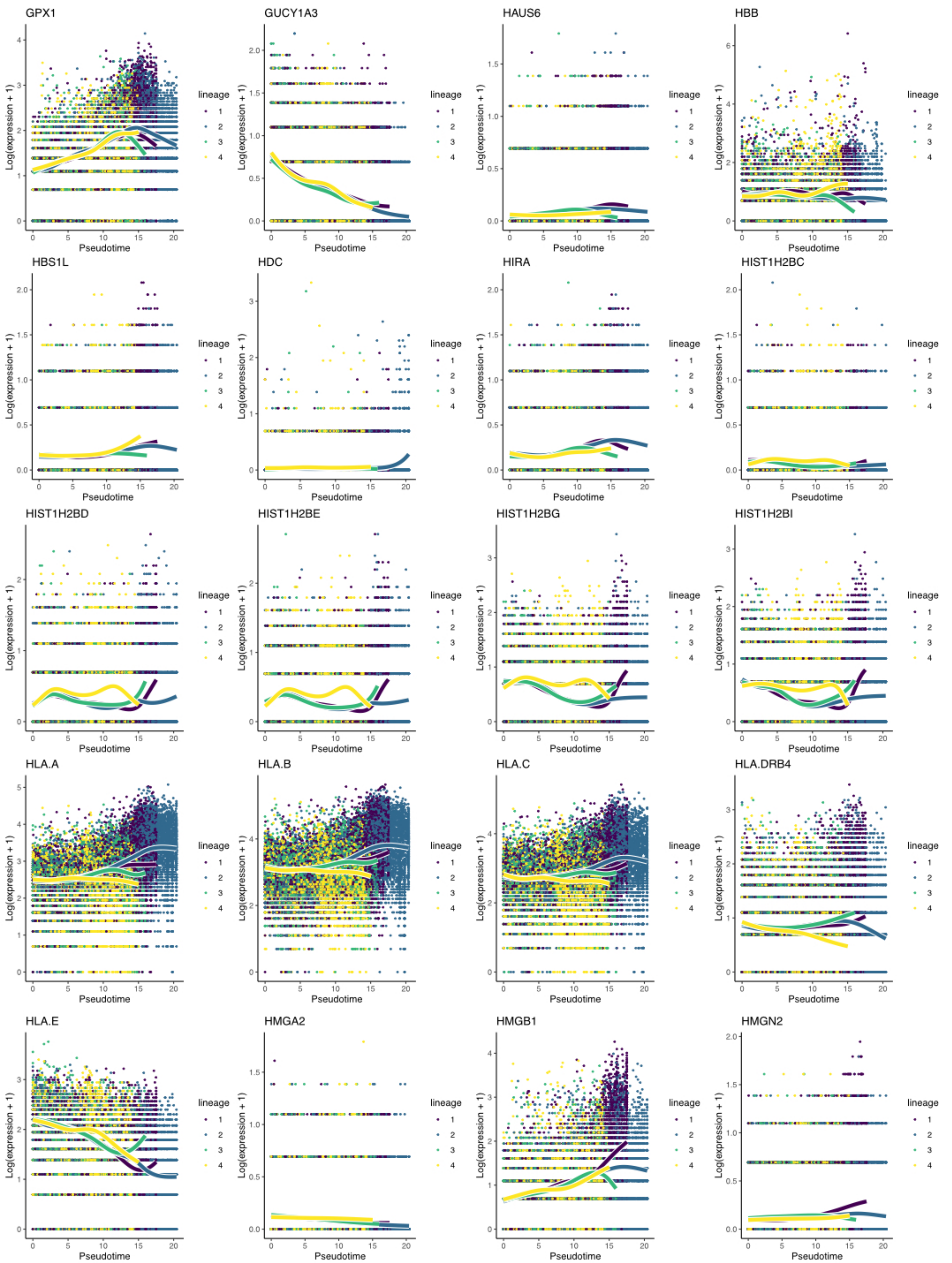

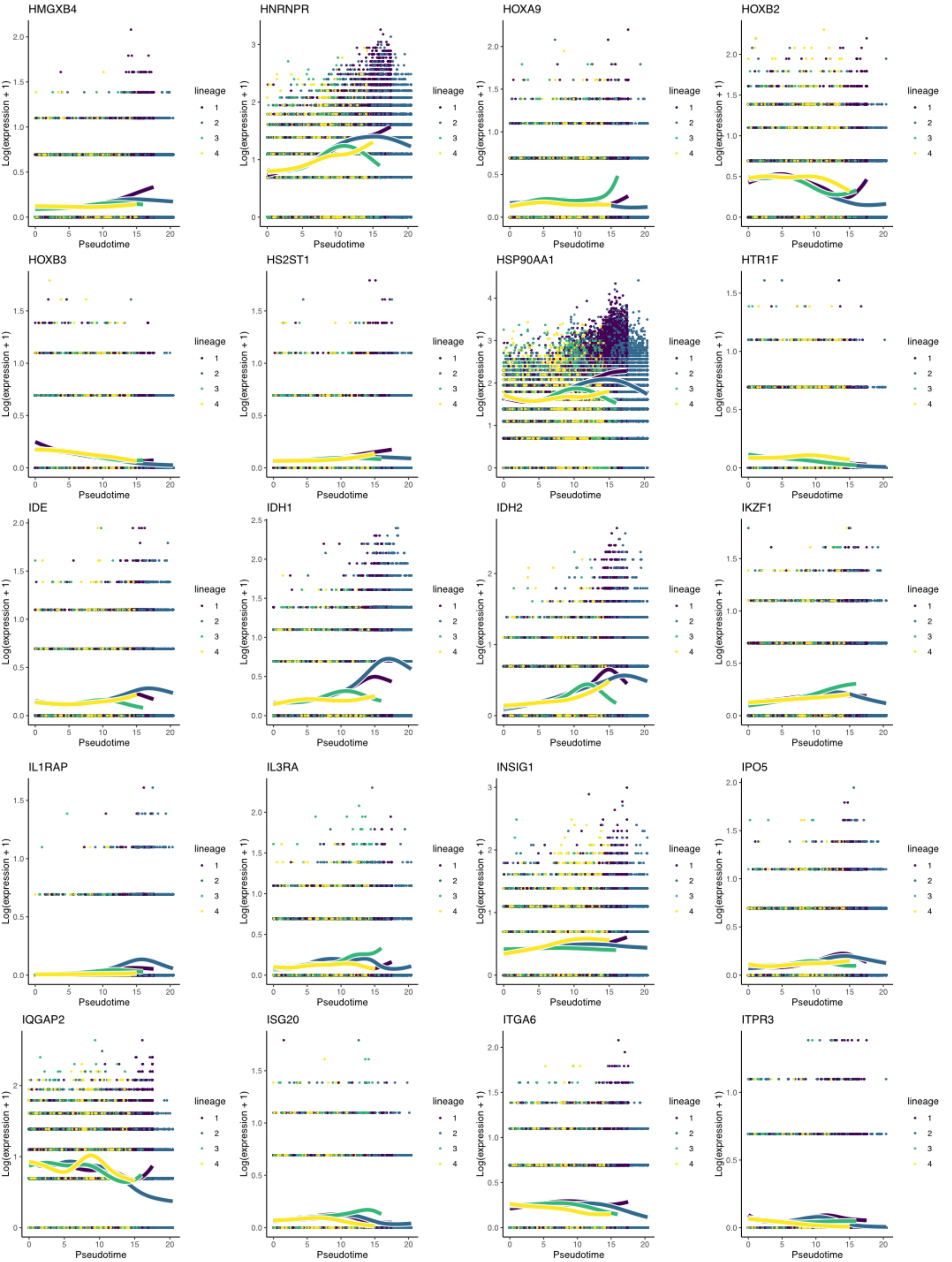

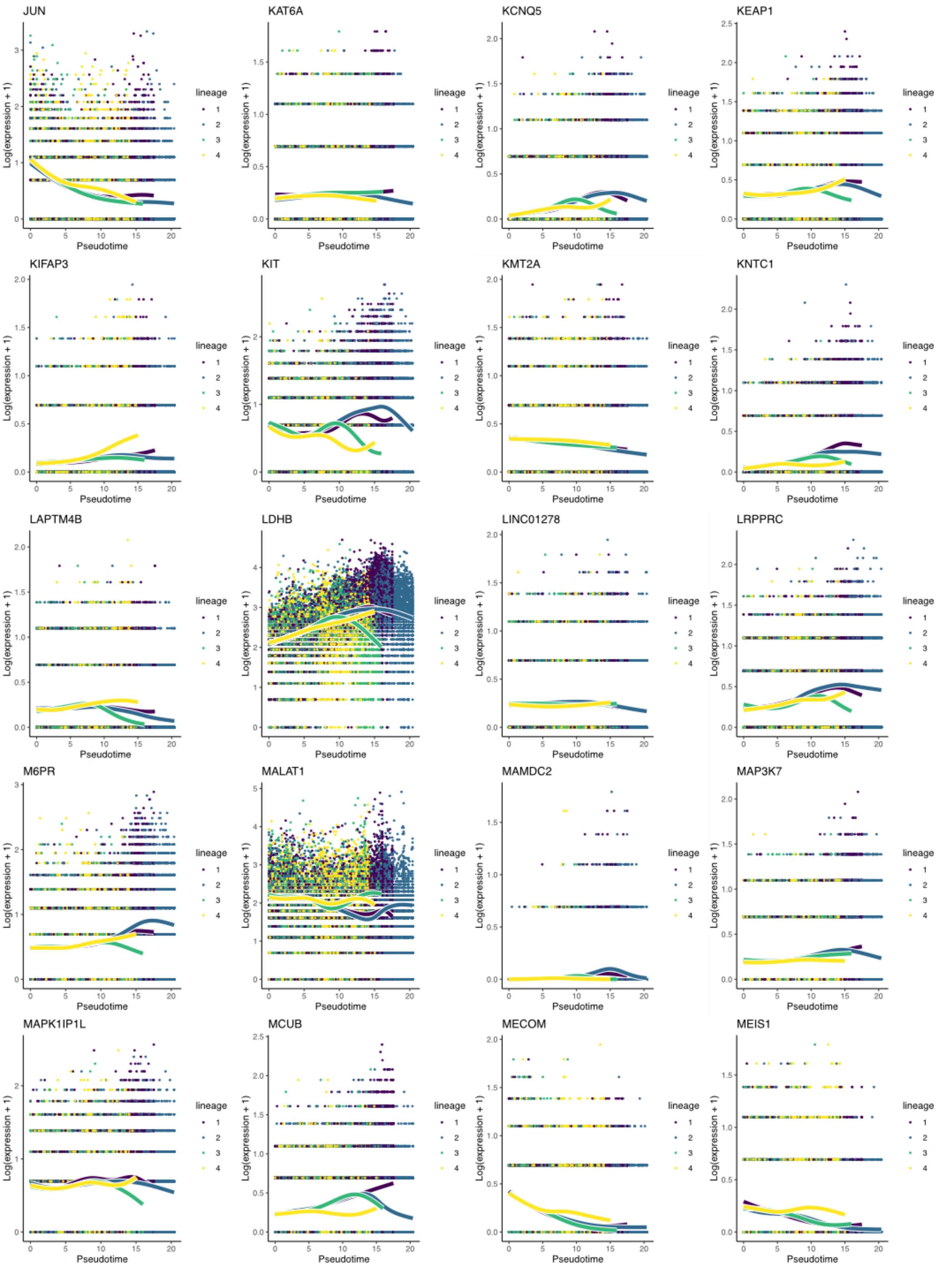

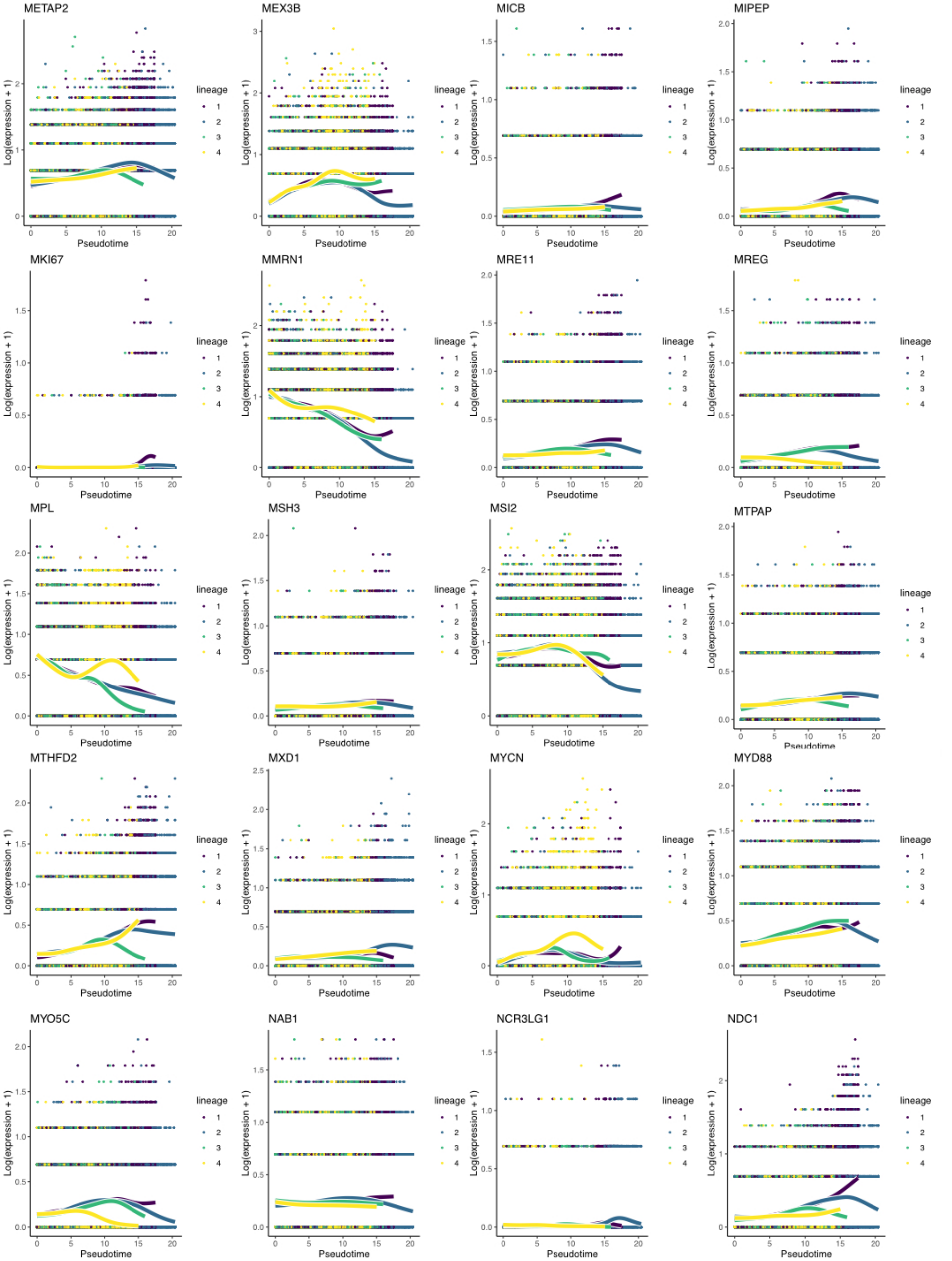

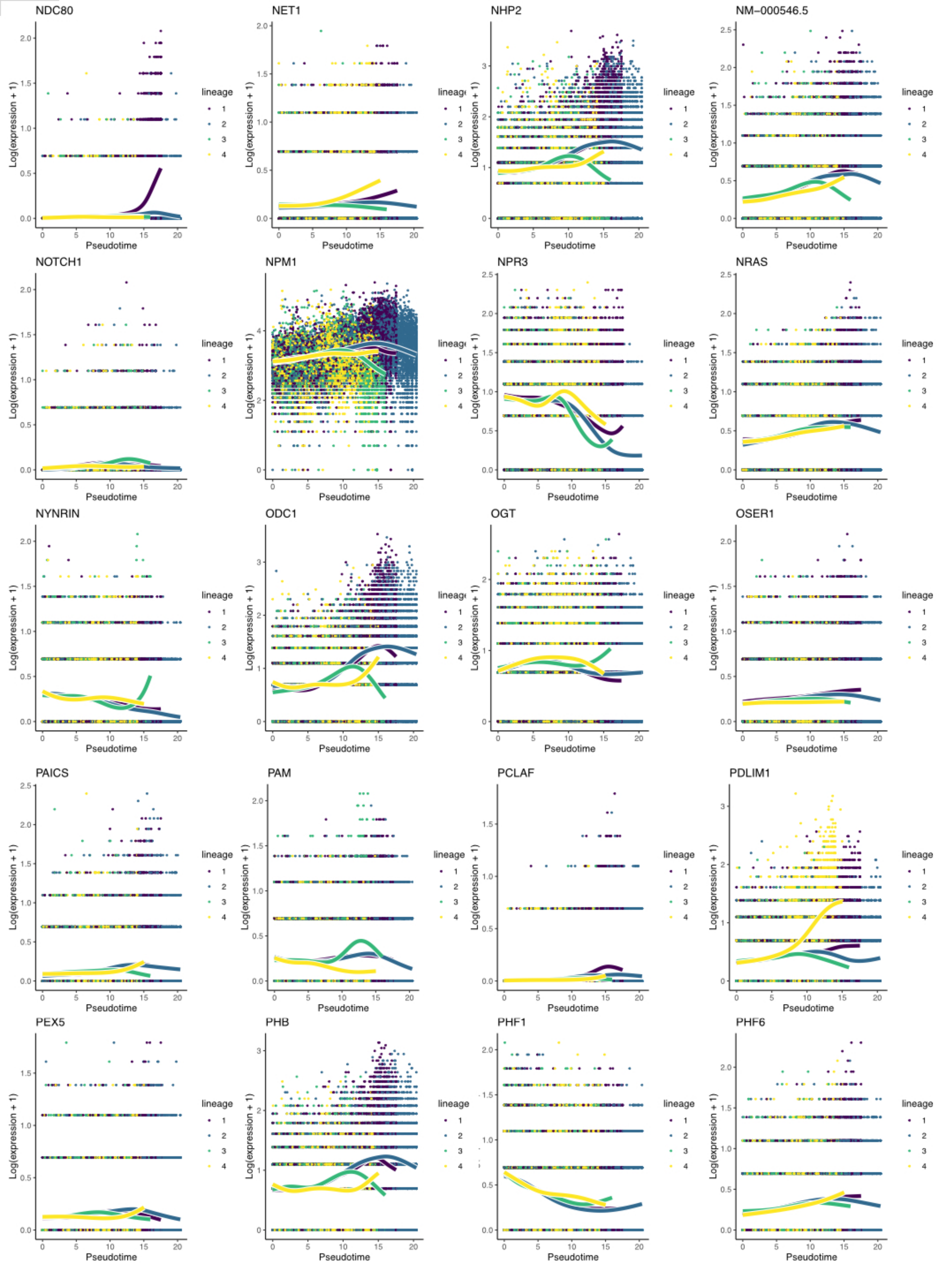

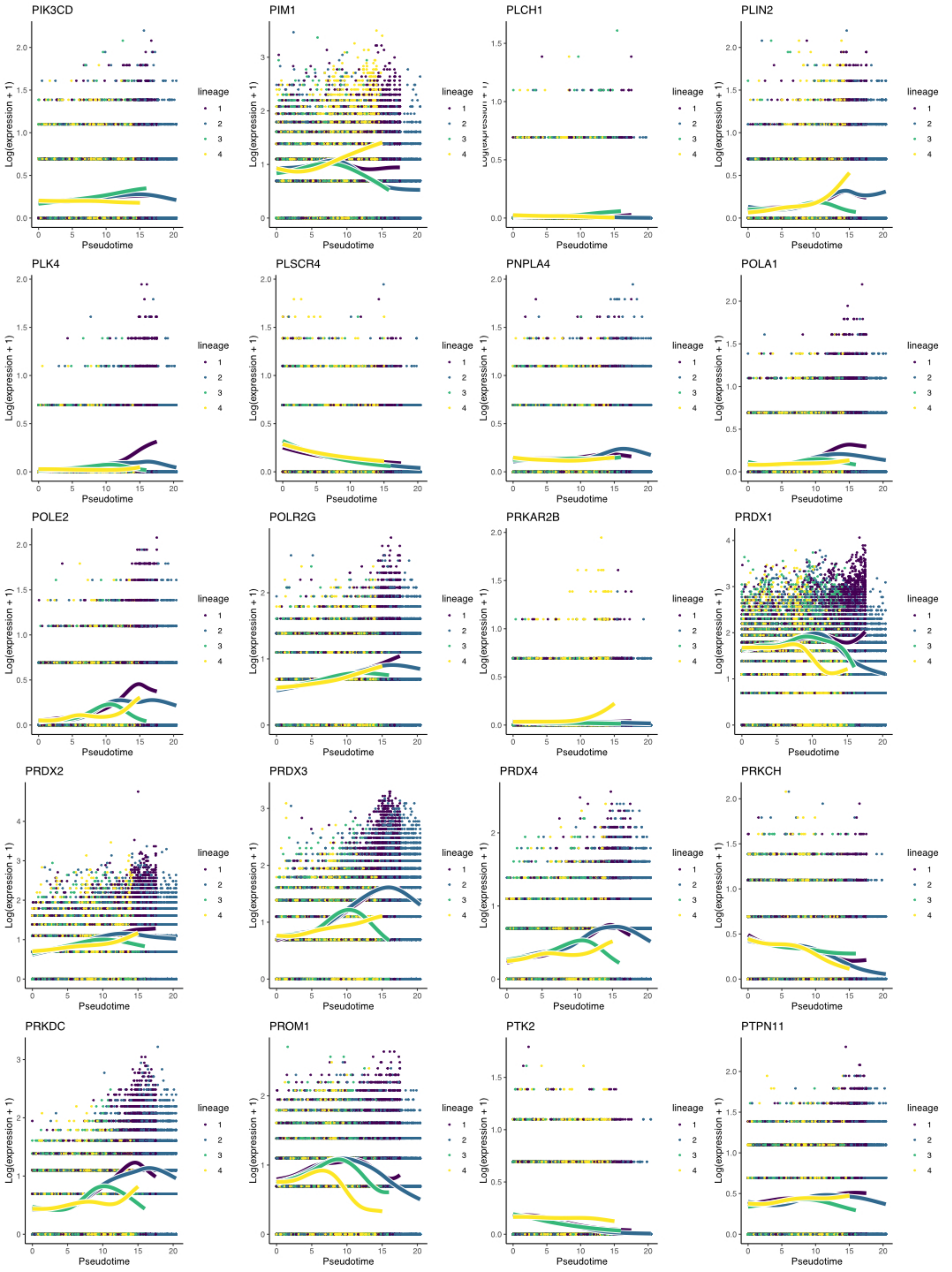

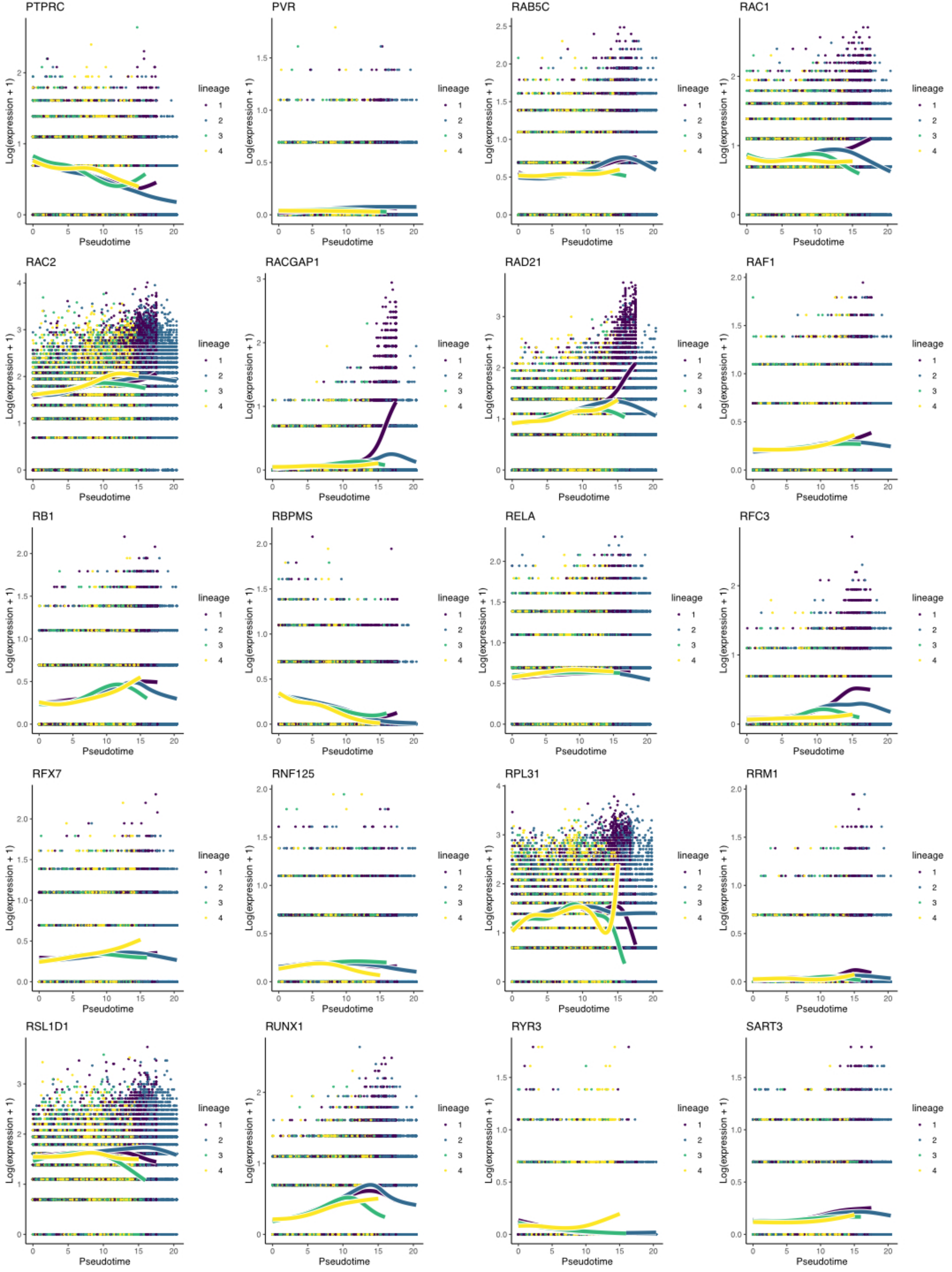

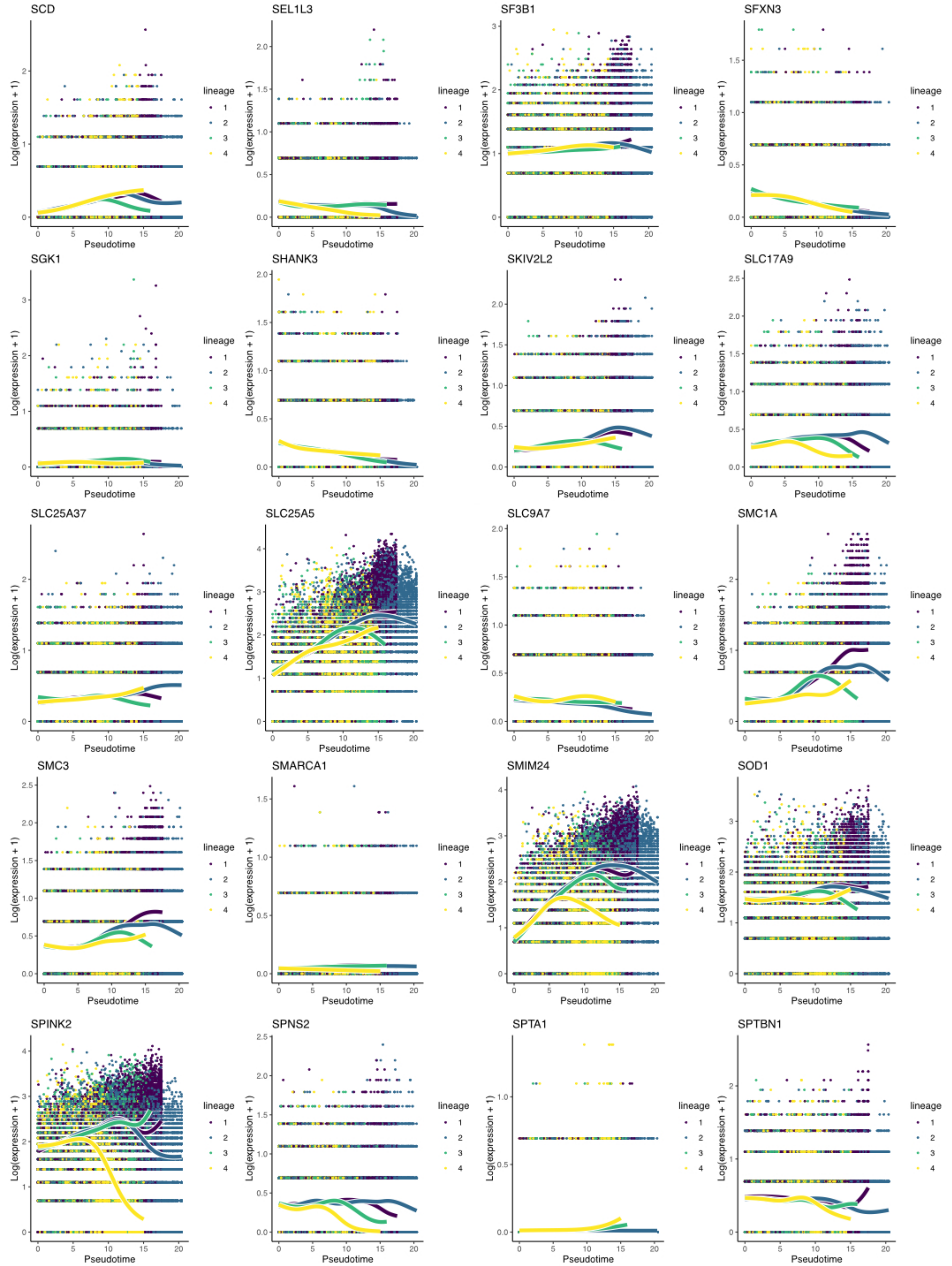

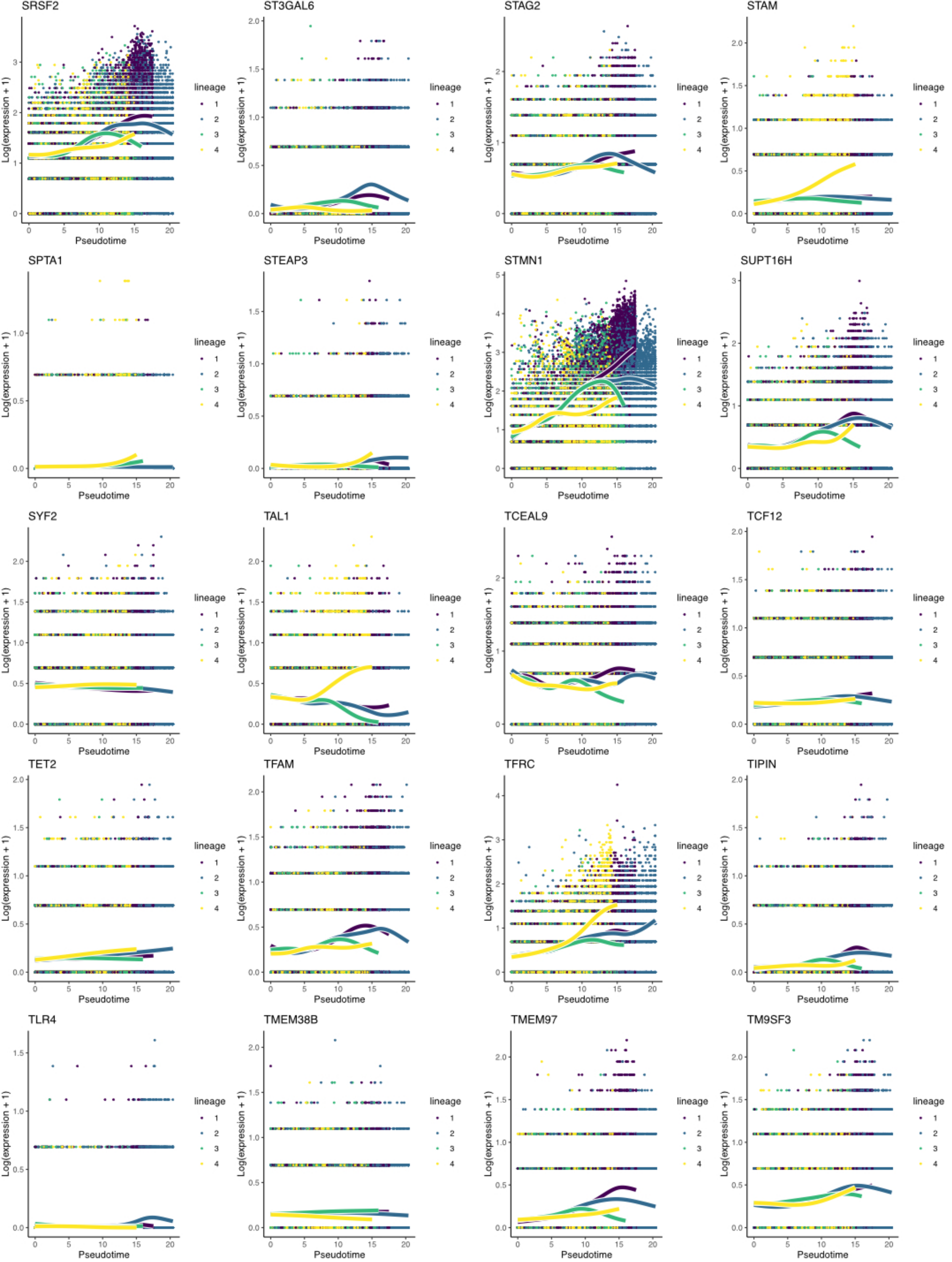

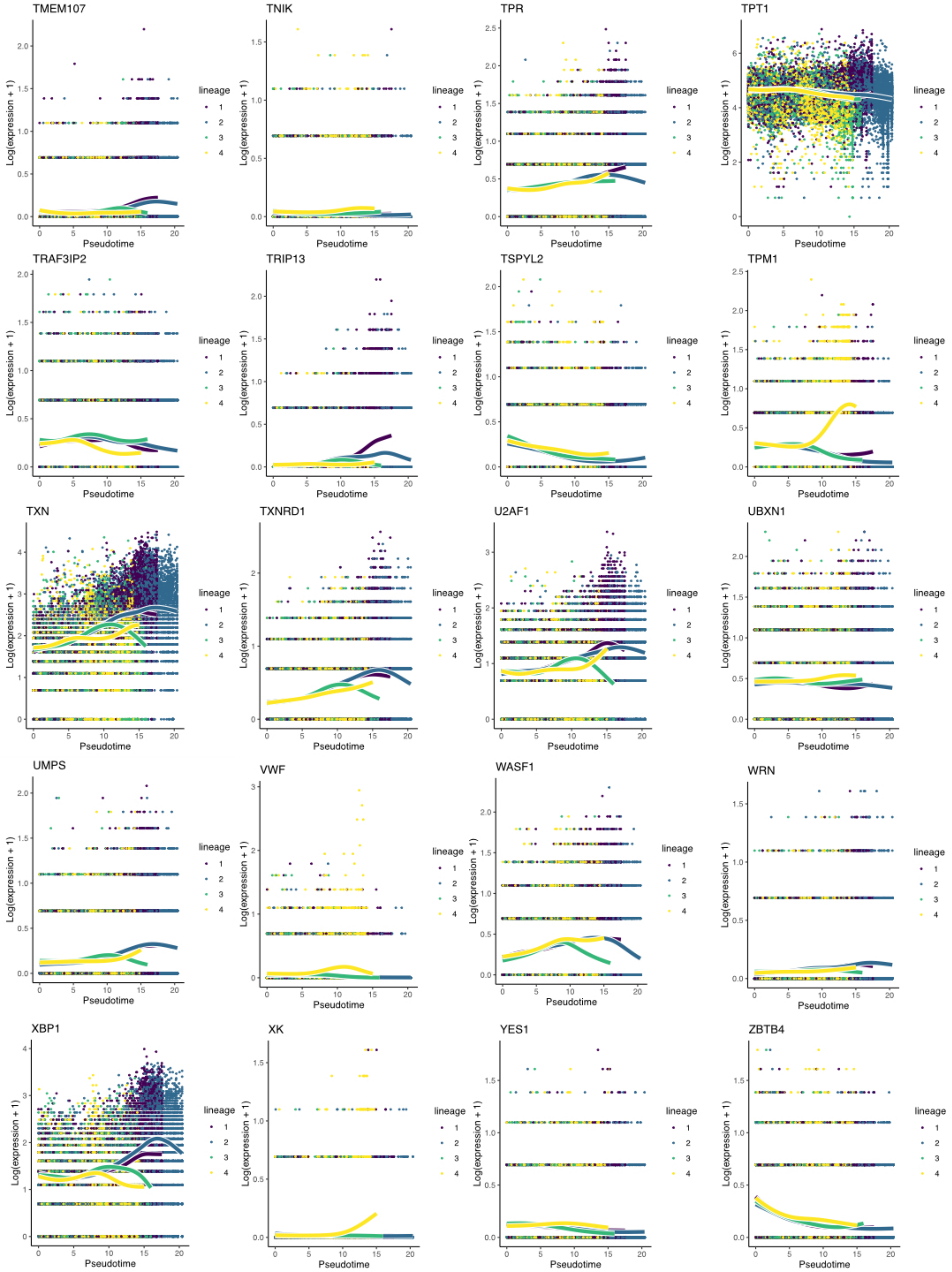

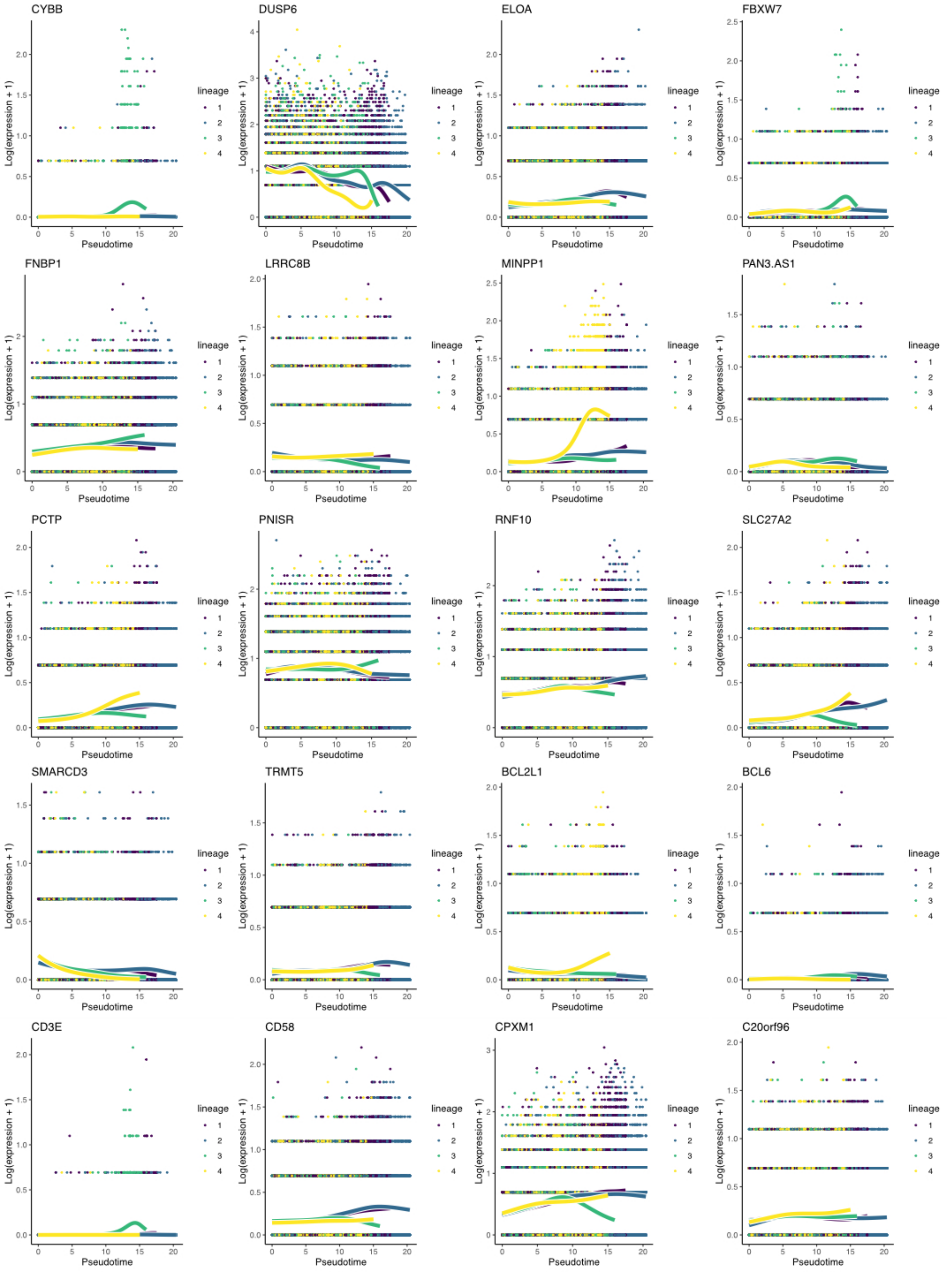

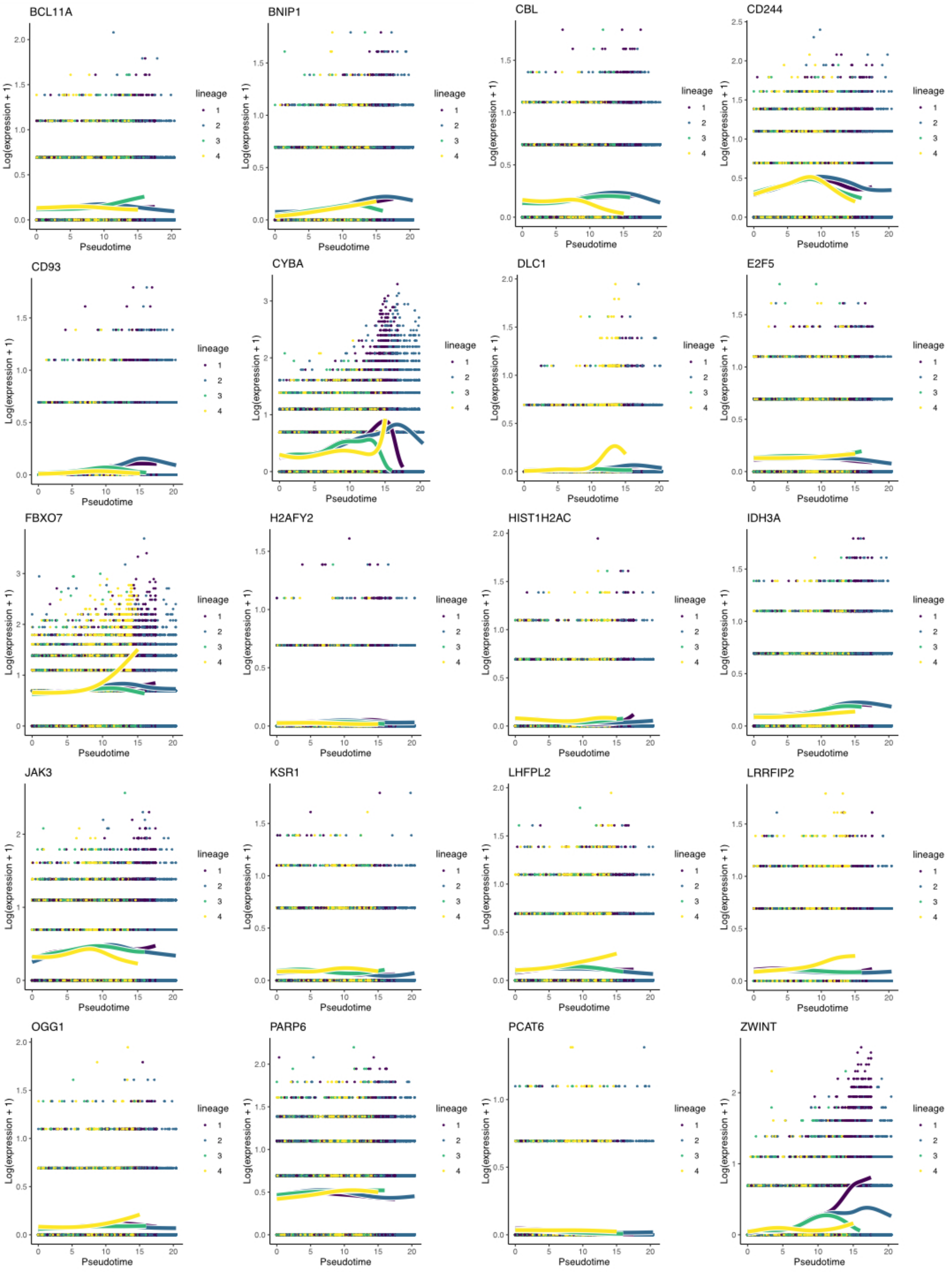

Supplement: Supplementary file 3 — Supplementary Data [file 41467_2025_57096_MOESM3_ESM.zip › supplementary data 13.pdf]
