# Supplementary material for: Continuous map of early hematopoietic stem cell differentiation across human lifetime
Source: Nat Commun. 2025 Mar 7;16:2287. doi: 10.1038/s41467-025-57096-y (PMC11889232; doi:10.1038/s41467-025-57096-y)
Supplement: Supplementary file 4 — Reporting Summary [file 41467_2025_57096_MOESM4_ESM.pdf]

nature portfolio

Corresponding author(s): Michael A. Rieger

Last updated by author(s): Jan 27, 2025

Reporting Summary

Nature Portfolio wishes to improve the reproducibility of the work that we publish. This form provides structure for consistency and transparency in reporting. For further information on Nature Portfolio policies, see our Editorial Policies and the Editorial Policy Checklist.

Statistics

For all statistical analyses, confirm that the following items are present in the figure legend, table legend, main text, or Methods section.

n/a	Confirmed	
☐	☒	The exact sample size (*n*) for each experimental group/condition, given as a discrete number and unit of measurement
☐	☒	A statement on whether measurements were taken from distinct samples or whether the same sample was measured repeatedly
☐	☒	The statistical test(s) used AND whether they are one- or two-sided<br>*Only common tests should be described solely by name; describe more complex techniques in the Methods section.*
☐	☒	A description of all covariates tested
☐	☒	A description of any assumptions or corrections, such as tests of normality and adjustment for multiple comparisons
☐	☒	A full description of the statistical parameters including central tendency (e.g. means) or other basic estimates (e.g. regression coefficient) AND variation (e.g. standard deviation) or associated estimates of uncertainty (e.g. confidence intervals)
☐	☒	For null hypothesis testing, the test statistic (e.g. *F*, *t*, *r*) with confidence intervals, effect sizes, degrees of freedom and *P* value noted<br>*Give P values as exact values whenever suitable.*
☐	☒	For Bayesian analysis, information on the choice of priors and Markov chain Monte Carlo settings
☐	☒	For hierarchical and complex designs, identification of the appropriate level for tests and full reporting of outcomes
☐	☒	Estimates of effect sizes (e.g. Cohen's *d*, Pearson's *r*), indicating how they were calculated

Our web collection on statistics for biologists contains articles on many of the points above.

Software and code

Policy information about availability of computer code

Data collection	R code is available at: https://github.com/TessaSchm/Early-HSC-differentiation/tree/main
Data analysis	R code is available at: https://github.com/TessaSchm/Early-HSC-differentiation/tree/main

For manuscripts utilizing custom algorithms or software that are central to the research but not yet described in published literature, software must be made available to editors and reviewers. We strongly encourage code deposition in a community repository (e.g. GitHub). See the Nature Portfolio guidelines for submitting code & software for further information.

Data

Policy information about availability of data

All manuscripts must include a data availability statement. This statement should provide the following information, where applicable:

- Accession codes, unique identifiers, or web links for publicly available datasets
- A description of any restrictions on data availability
- For clinical datasets or third party data, please ensure that the statement adheres to our policy

Proteo-Transcriptomic single cell sequencing data is available online in the ArrayExpress repository via accession link: https://www.ebi.ac.uk/biostudies/arrayexpress/studies/E-MTAB-14596?key=6eb62c54-2e2a-466e-bc78-a2c690645830. Source Data are provided with this paper.

Research involving human participants, their data, or biological material

Policy information about studies with human participants or human data. See also policy information about sex, gender (identity/presentation), and sexual orientation and race, ethnicity and racism.

Reporting on sex and gender	We report on the sex of the donors of biological samples. Our study did not seperate between sex. The donors are listed in Supplementary Tables 3 and 15.
Reporting on race, ethnicity, or other socially relevant groupings	not provided, because this information was not reported
Population characteristics	Proteo-Transcriptomic single cell sequencing: We included 15 BM samples in our study, five of young age (20-23 years old), six of middle age (52-65 years old) and four of old age (70-84 years old). In total, there were eight male and seven female participants, with equal distribution in age groups.<br>Functional validation studies: Donor distribution included 27 males and 6 females. The average age of the healthy donors was 33 years. Epidemiology was consistent with the typical demographics of a German donor registry.
Recruitment	no self-selection bias on the recruitment of human samples, samples were provided by the respective University Hospitals as indicated in the Methods.
Ethics oversight	Ethical permits for the study: 011-17 University of Gothenburg, #329/10 University of Frankfurt and 2019.143 University of Pamplona

Note that full information on the approval of the study protocol must also be provided in the manuscript.

Field-specific reporting

Please select the one below that is the best fit for your research. If you are not sure, read the appropriate sections before making your selection.

- [x] Life sciences
- [ ] Behavioural & social sciences
- [ ] Ecological, evolutionary & environmental sciences

For a reference copy of the document with all sections, see nature.com/documents/nr-reporting-summary-flat.pdf

Life sciences study design

All studies must disclose on these points even when the disclosure is negative.

Sample size	Sample size was estimated by a power calculation.
Data exclusions	No data were excluded.
Replication	Number of replications indicated in the figure legends.
Randomization	Random allocations for all experiments.
Blinding	Investigators were blined during data acquisition and analysis whenever possible or necessary.

Reporting for specific materials, systems and methods

We require information from authors about some types of materials, experimental systems and methods used in many studies. Here, indicate whether each material, system or method listed is relevant to your study. If you are not sure if a list item applies to your research, read the appropriate section before selecting a response.

Materials & experimental systems

n/a	Involved in the study	
[ ]	[x]	Antibodies
[x]	[ ]	Eukaryotic cell lines
[x]	[ ]	Palaeontology and archaeology
[x]	[ ]	Animals and other organisms
[x]	[ ]	Clinical data
[x]	[ ]	Dual use research of concern
[x]	[ ]	Plants

Methods

n/a	Involved in the study	
[x]	[ ]	ChIP-seq
[ ]	[x]	Flow cytometry
[x]	[ ]	MRI-based neuroimaging

Antibodies

Antibodies used	see all antibodies used for the study (CITE-seq, FACS, functional assays, WB) listed in Suppl. Tables 2 and 16
Validation	all antibodies used have been extensively tested and validated by the manufacturers and cited in the litaerature.

Plants

Seed stocks	not applicable
Novel plant genotypes	not applicable
Authentication	not applicable

Flow Cytometry

Plots

Confirm that:

- [x] The axis labels state the marker and fluorochrome used (e.g. CD4-FITC).
- [x] The axis scales are clearly visible. Include numbers along axes only for bottom left plot of group (a 'group' is an analysis of identical markers).
- [x] All plots are contour plots with outliers or pseudocolor plots.
- [x] A numerical value for number of cells or percentage (with statistics) is provided.

Methodology

Sample preparation	see Method section
Instrument	BD FACS Celesta, BD FACSAria II, BD FACSAria III, BD FACS Fortessa
Software	BD FACS Diva 8.0.1 and BD FlowJo Software 10.9.0
Cell population abundance	reanalysis of sorted cells, purity of >95% was achieved in all experiments
Gating strategy	see method section and figures/supplementary figures

- [x] Tick this box to confirm that a figure exemplifying the gating strategy is provided in the Supplementary Information.
